# ^1^H NMR Urinary Metabolomic Analysis in Older Adults after Hip Fracture Surgery May Provide Valuable Information for Patient Profiling—A Preliminary Investigation

**DOI:** 10.3390/metabo12080744

**Published:** 2022-08-12

**Authors:** Wafa Douzi, Delphine Bon, Sara Suikkanen, Paula Soukkio, Nadège Boildieu, Arja Nenonen, Markku Hupli, Katriina Kukkonen-Harjula, Benoit Dugué

**Affiliations:** 1 Laboratoire «Mobilité, Vieillissement, Exercice (MOVE)—UR 20296», Faculté des Sciences du Sport, Université de Poitiers, 8 Allée Jean Monnet, 86000 Poitiers, France; 2 INSERM U1313, (IRMETIST), Poitiers, France and Faculty of Medicine and Pharmacy, University of Poitiers, 86000 Poitiers, France; 3 Faculty of Sport and Health Sciences, University of Jyväskylä, 40014 Jyväskylä, Finland; 4 Faculty of Social Services and Health Care, LAB University of Applied Sciences, 53130 Lappeenranta, Finland; 5 Rehabilitation and Laboratory Center, South Karelia Social and Health Care District (Eksote), Valto Käkelän katu 3, 53130 Lappeenranta, Finland

**Keywords:** hip fracture, frailty, functioning, metabolomics, nuclear magnetic resonance spectroscopy

## Abstract

In these times of precision and personalized medicine, profiling patients to identify their needs is crucial to providing the best and most cost-effective treatment. In this study, we used urine metabolomics to explore the characterization of older adults with hip fractures and to explore the forecasting of patient outcomes. Overnight urine specimens were collected from 33 patients (mean age 80 ± 8 years) after hip fracture surgery during their stay at a rehabilitation hospital. The specimens were analyzed with ^1^H NMR spectroscopy. We performed a metabolomics study regarding assessments of frailty status, Functional Independence Measure (FIM), and Short Physical Performance Battery (SPPB). The main metabolic variations concerned 10 identified metabolites: paracetamol derivatives (4 peaks: 2.15 ppm; 2.16 ppm; 7.13 ppm and 7.15 ppm); hippuric acid; acetate; acetone; dimethylamine; glycine; alanine; lactate; valine; TMAO. At baseline, the urinary levels of these metabolites were significantly higher (i) in frail compared with non-frail patients, (ii) in persons with poorer FIM scores, and (iii) in persons with poorer compared SPPB scores. Our findings suggested that patients with increased levels of urine metabolites associated with metabolic, inflammatory, and renal disorders presented clear signs of frailty, impaired functional independence, and poor physical performance. Metabolomics could be a valuable tool to further characterize older adults, especially after major medical events.

## 1. Introduction

Providing the best available and most cost-effective interventions is a key issue in the context of the prevention, treatment, and rehabilitation of diseases. In these times of precision and personalized medicine, patient profiling to identify specific needs is crucial. Thus, interventions can be tailored to target specific outcomes according to the individual capacities or even to the individual abilities to be improved. Clearly, it is not a simple task to phenotype persons according to their level of functioning and their capacity to improve. Nevertheless, personalized interventions designed for disabled older adults with specific capacities could theoretically enable them to efficiently improve autonomy and functioning.

In the context of aging, characterizing older persons according to signs of frailty has gained importance during the last 20 years. For instance, the assessment of frailty using valid instruments (e.g., Fried’s frailty phenotype criteria) [1] has allowed health care professionals to evaluate patients’ risk factors before a loss of autonomy. Therefore, it could be possible to plan a tailored intervention against risk factors, such as biological, psychological, behavioral, social, and environmental factors, to provide improvements in motor skills, cognitive functions, and autonomy [2,3]. Nevertheless, the characterization of frailty is usually binary (yes/no; high risk/low risk) and does not reflect the complexity and the dynamic nature of frailty. During frailty development, subclinical signs emerge, but they are difficult to detect. However, these subclinical signs can quickly develop into clear signs when a dramatic event occurs (e.g., falls, infections), reflecting the vulnerability and decreasing resources of the older person. Metabolic abnormalities after a fall can predict short-term mortality in older patients [4]. Therefore, it would be of great importance to detect early signs of metabolic dysregulation associated with frailty [5]. Hip fracture surgery in older adults offers a model to investigate metabolic dysregulation. Osteoporotic hip fractures are a major public health problem because of their adverse effects such as substantial disability and increased mortality risk. In the United States, a quarter of a million hip fractures cost more than 8 billion dollars each year, for acute medical care and nursing home services. Future costs will be even higher as hip fracture incidence rates increase worldwide: by the year of 2050, there will be more than 4.5 million hip fractures [6]. Hip fracture treatment is expensive (cost estimation per year in Europe: 16 billion euros) [7] and rehabilitation is not always successful. Effective prevention of falls may alleviate the enormous social burden of hip fractures. Thus, metabolomic analysis could be applied to investigate metabolic profiles in older persons and to identify underlying health problems associated with frailty or age-related diseases. Previous studies have shown that the presence of some serum metabolites or dysregulated molecules in preoperative hip fracture patients is associated with complications during the postoperative period [8,9,10].

Modern technologies combined with big data management, such as metabolomics, provide new possibilities for investigating changes in metabolic states during aging and age-associated diseases or disability [11,12,13]. Such technologies could contribute to the identification of biomarkers or metabolites related to human aging and frailty development [11,12,13,14]. Targeted metabolomics analysis using ^1^H nuclear magnetic resonance (^1^H NMR) spectroscopy also provides a comprehensive analysis of metabolite profiles and furnishes key information for the further understanding of related metabolic pathways and their changes during aging and frailty processes. ^1^H NMR spectroscopy is sometimes considered a not very sensitive method but it is a robust and reproducible one compared to other techniques such as gas or liquid chromatography combined with mass spectrometry. Furthermore, the NMR technique does not require any sample preparation (except pH adjustment) [15,16].

In this study, urine metabolomics with ^1^H NMR spectroscopy was used to investigate the characterization of patients with hip fractures who underwent surgical repair and to explore the forecasting of patient outcomes. Among our patients, we analyzed the associations between each patient’s baseline urine metabolome and frailty severity, and their Functional Independence Measure (FIM) and Short Physical Performance Battery (SPPB) scores. We investigated whether urinary metabolomics of specimens collected in the rehabilitation hospital after surgery could provide relevant patterns to predict patient outcomes, such as their performance in FIM and SPPB one year later, as well as patient survival during the follow-up of 2 years. This work is an ancillary substudy of the randomized HIPFRA study where patients participated in year-long home-based exercise training [17].

## 2. Results

### 2.1. Participant Description

Thirty-three patients participated in the study (26 women and 7 men, age 80 ± 8 years). Their characteristics are described in Table 1. Age, BMI, scores of frailty, SPPB, and FIM (total, motor, and cognitive) are shown for all participants and according to their frailty status (non-frail, pre-frail, and frail). Frail participants had, as expected, a higher score of frailty compared to non-frail (*p* < 0.05) and pre-frail (*p* < 0.05) participants. For other parameters, we did not observe any significant differences among these subgroups.

### 2.2. Description of the Urine Bank Spectrum

#### 2.2.1. Unsupervised Multivariate Analysis

We applied a principal component analysis (PCA) to the urine spectra using a bucket of 0.05 ppm. In the score plot, one spectrum was detected as an outlier. The loading and contribution plots indicate that this outlier differs from the others because of a large contribution of the peak for acetate compared with all the other spectra (Supplemental Figure S1). However, we decided to keep it in future analyses. With this first analysis, we selected peaks in the bucket with a score contribution greater than 0.5 or less than −0.5. We retained 18 peaks that were identified in the literature.

#### 2.2.2. Identification of Metabolites of Interest in Urine

**Figure 1:**
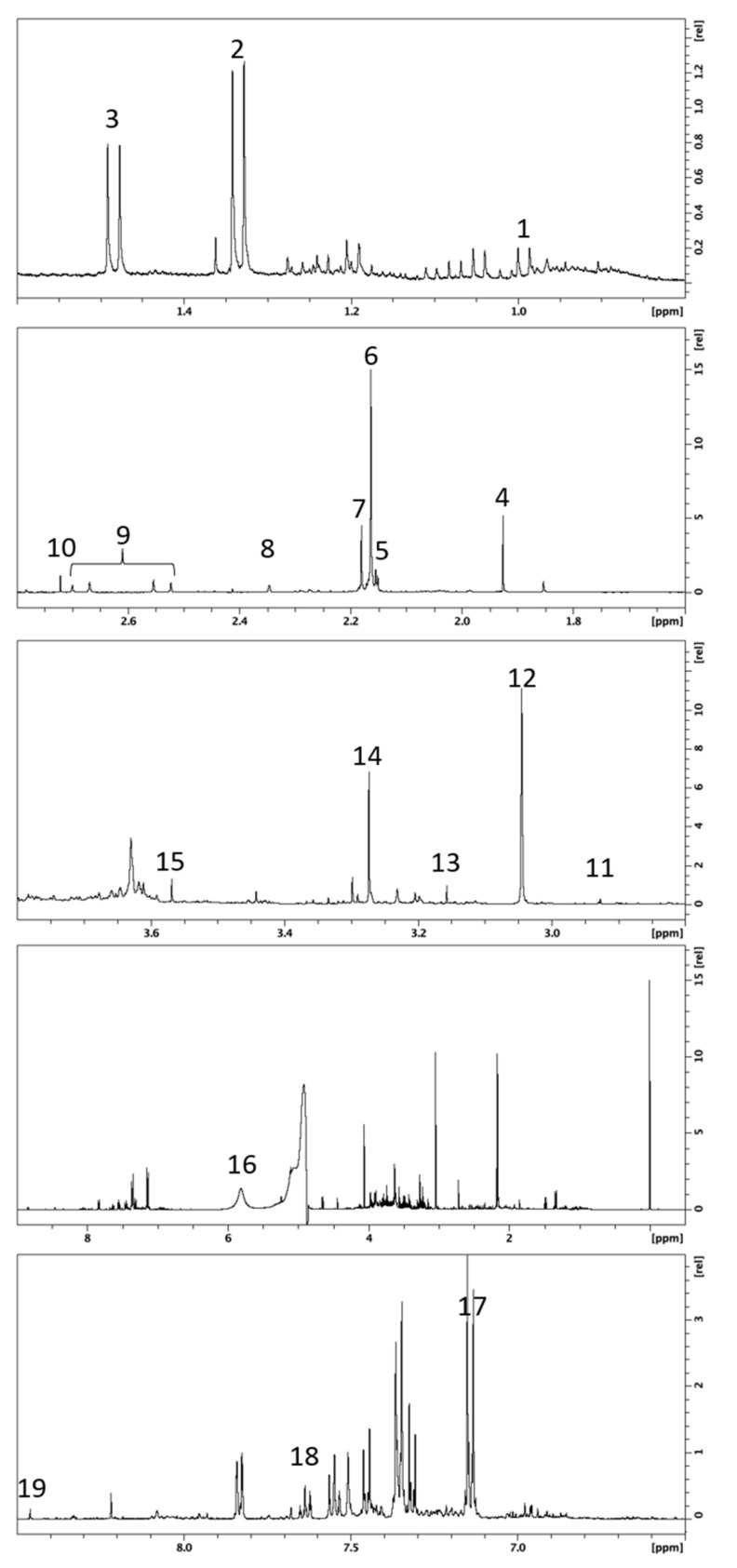
Typical 500 MHz ^1^H NMR spectrum from one urine sample. Numbers refer to assigned metabolites presented in Table 2.

Figure 1 is a representative ^1^H NMR spectrum from one urine sample. The metabolites that showed interesting variabilities between all samples are presented in Table 2. We found three amino acids (alanine, valine, and glycine), and one ketone (acetone). Organic acids were also of interest, such as lactic acid, acetic acid, hippuric acid, formic acid, and citric acid. Some nitrogen derivatives complete the list: dimethylamine, methylguanidine, creatinine, dimethyl sulfone, trimethylamine-N-oxide (TMAO), and urea. Finally, some exogenous molecules were also found in the patient’s urine: derivatives from paracetamol (including acetaminophen glucuronide and acetaminophen sulfate), as well as hippuric acid, which can also be endogenous.

### 2.3. Outcome Analyses

In the following section, we present outcome analyses, including frailty status, FIM, SPPB, and mortality. Sex and age comparisons are presented in the Supplementary File (Supplementary Figures S2 and S3).

#### 2.3.1. Frailty

**Figure 2:**
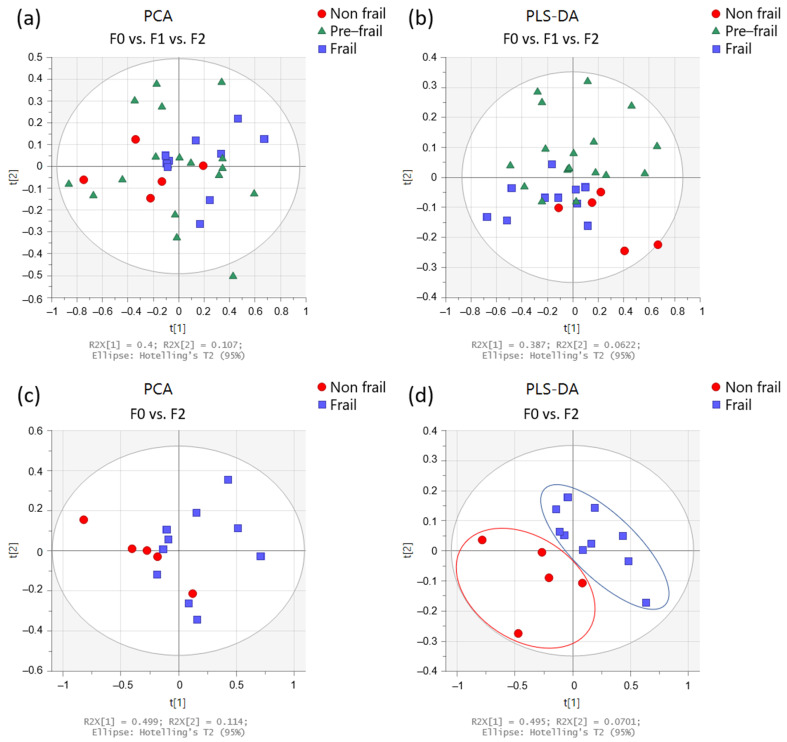
Multivariate analysis obtained from urine spectra of the participants (n = 33). Score plots from PCA (**left** panels) and PLS-DA (**right** panels) according to frailty severity: (**a**,**b**) in non-frail (red circles; n = 5), pre-frail (green triangles; n = 18), and frail (blue squares; n = 10) participants, and (**c**,**d**) in non-frail (red circles) and frail (blue squares) participants.

Figure 2 shows multivariate analyses of the urine spectrum when comparing patients with varying frailty severity (non-frail, pre-frail, and frail). On the score plots from PCA and PLS-DA (partial least square—discriminant analysis) obtained from all patients, we can observe a trend: the non-frail and frail spots seem to be on opposite sides in the PC1 dimension, while the pre-frail spots are scattered throughout the graph (Figure 2a). When comparing only non-frail and frail patients (Figure 2d), better separation is observed.

**Figure 3:**
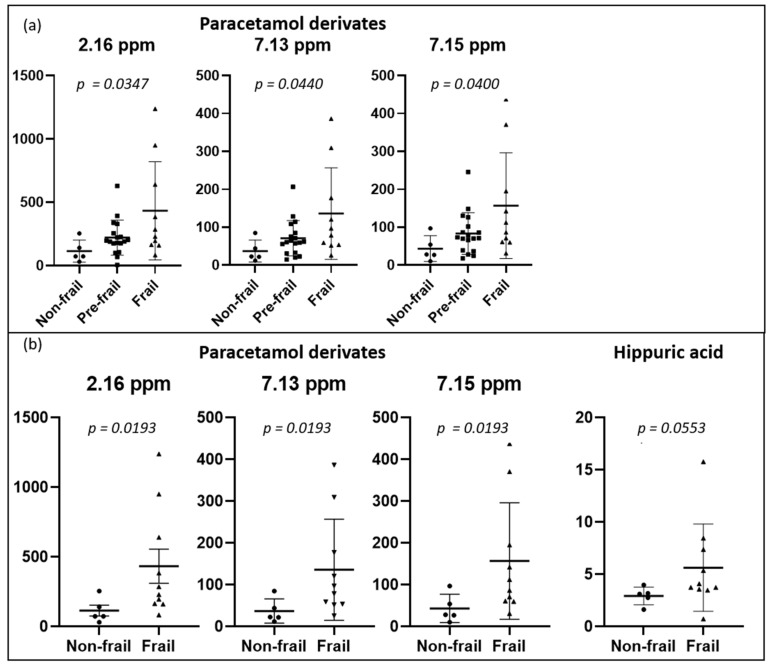
Dot plots of targeted metabolites showing significant differences between groups, (**a**) non-frail (n = 5) vs. pre-frail (n = 18) vs. frail (n = 10) participants, and (**b**) non-frail vs. frail participants. Plots represent mean ± standard deviation.

We also performed a classical integration analysis targeting our 19 peaks using an ANOVA test to compare the three groups (non-frail, pre-frail, and frail) (Figure 3a). When the *p*-value was lower than 0.05, a posthoc test (Tukey) was applied to perform multiple comparisons between the groups. These results revealed a trend toward a difference between the non-frail and frail groups. As presented in Figure 3b, a Mann–Whitney test was also performed to assess the difference between the non-frail and frail groups, and the level of paracetamol derivatives was significantly higher in frail patients. Regarding hippuric acid, we observed a tendency toward higher values in frail patients.

#### 2.3.2. Functional Independence Measure (FIM)

**Figure 4:**
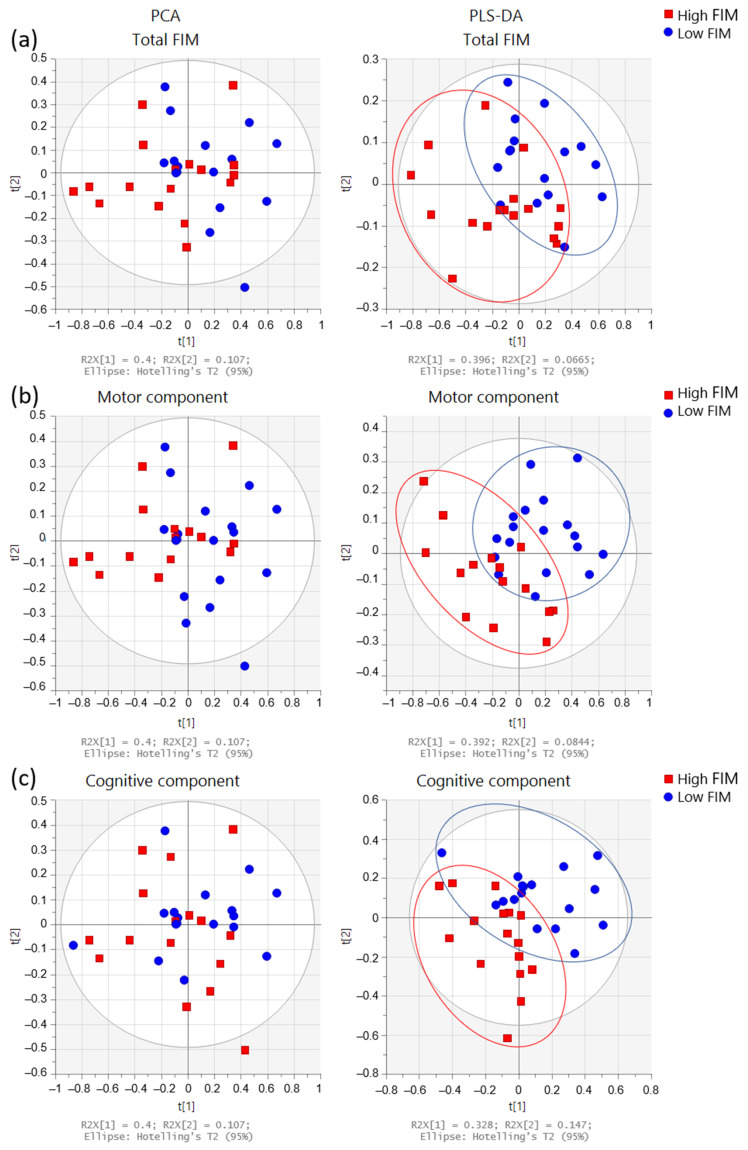
Multivariate analysis obtained from the urine spectra of the participants (n = 33). Score plots from the PCA (**left** side) and PLS-DA (**right** side) for FIM scores: Comparisons between (**a**) total FIM: low ≤102 (n = 16) and high >102 (n = 17); (**b**) motor component: low ≤74 (n = 18) and high >74 (n = 15), and (**c**) cognitive component: low ≤31 (n = 17) and high >31 (n = 16). FIM groups were dichotomized by medians: high FIM (red squares) and low FIM (blue circles).

**Figure 5:**
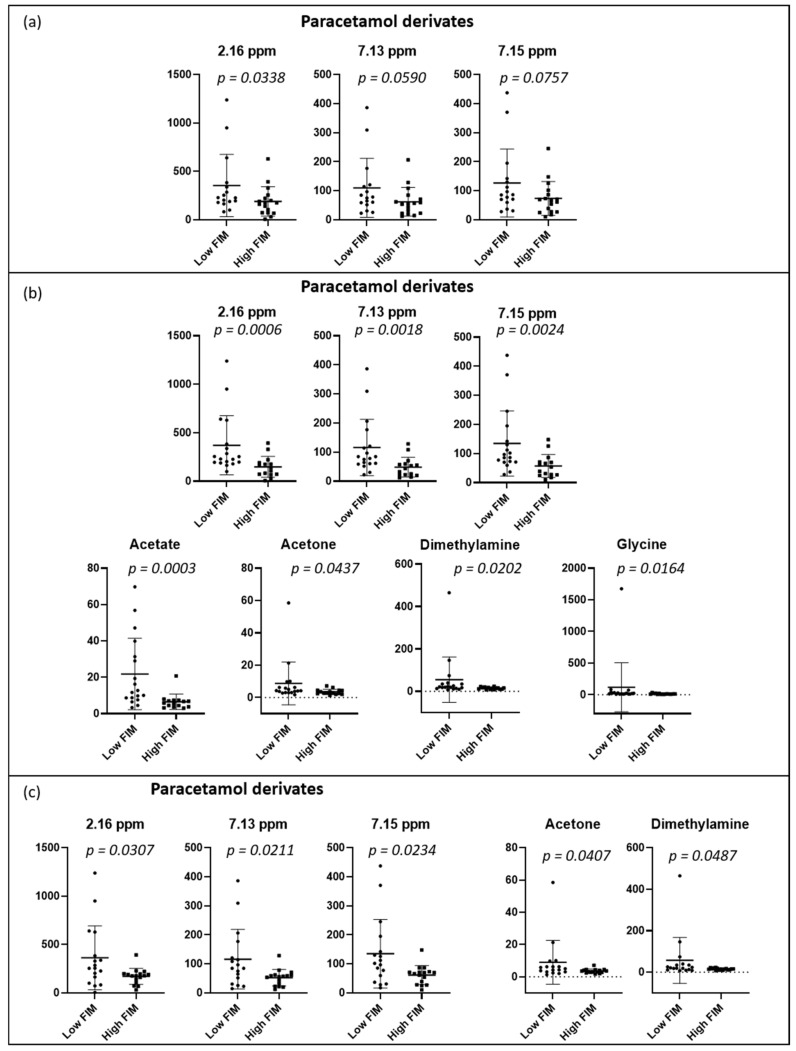
Dot plots of targeted metabolites showing significant differences between groups, (**a**) total FIM: low ≤102 (n = 16) and high >102 (n = 17); (**b**) motor component: low ≤74 (n = 18) and high >74 (n = 15), and (**c**) cognitive component: low ≤31 (n = 17) and high >31 (n = 16). FIM groups were dichotomized with medians. Plots represent mean ± standard deviation.

PCA and PLS-DA of the urine spectrum to compare FIM scores (total, motor, and cognitive) are presented in Figure 4. When comparing the groups with low and high total FIM scores (dichotomized by medians), we can observe a slight discrimination. However, when we compare either the motor or the cognitive component, the separation appears to be better. To understand these separations, we conducted a targeted analysis. As shown in Figure 5a, we observed an increased level of paracetamol derivatives in groups with lower scores of total, motor, and cognitive FIM. Regarding the motor component (Figure 5b), the group with lower scores (FIM ≤ 74; poorer functional independence) presented higher levels of acetate, acetone, dimethylamine, and glycine than the group with higher scores (FIM > 74; better functional independence). Regarding the cognitive component (Figure 5c), we noticed higher levels of acetone and dimethylamine in the group with lower scores (FIM ≤ 31).

#### 2.3.3. Short Physical Performance Battery (SPPB)

**Figure 6:**
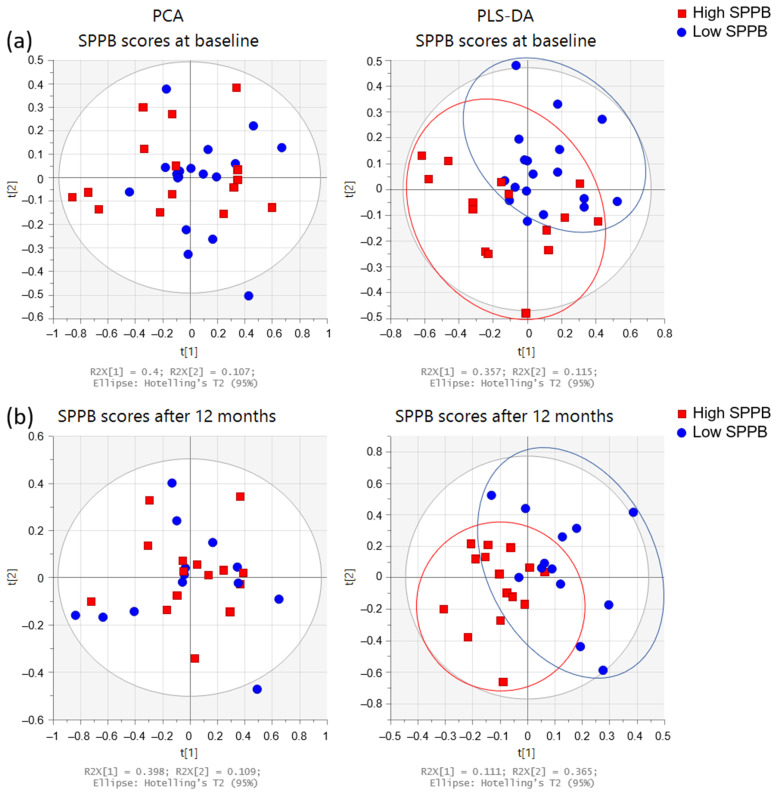
Multivariate analysis of the urine spectra of the participants’ SPPB (n = 33). Score plots from PCA (**left** side) and PLS-DA (**right** side): (**a**) at baseline (low ≤3 (n = 18); high >3 (n = 15)), and (**b**) after 12 months (low ≤8 (n = 13); high >8 (n = 15)). SPPB groups were dichotomized with medians: high SPPB (red squares) and low SPPB (blue circles).

**Figure 7:**
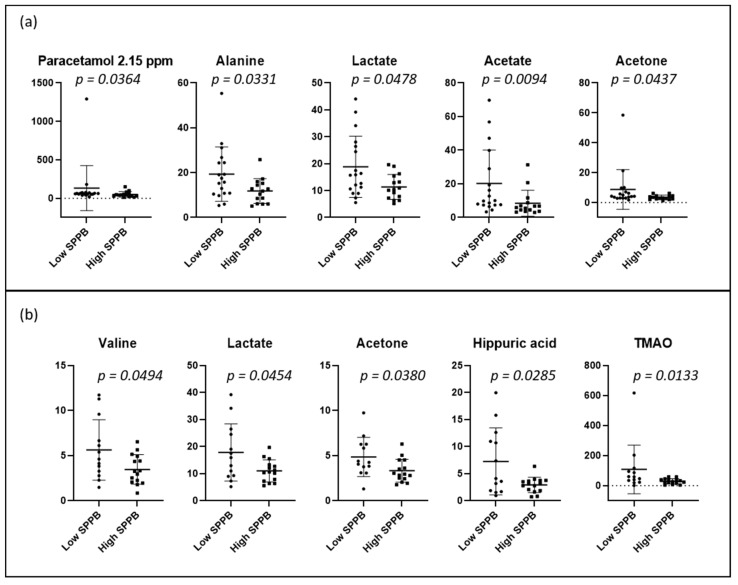
Dot plots of targeted metabolites showing significant differences between groups of high and low SPPB: (**a**) at baseline (low ≤3 (n = 18); high >3 (n = 15)), and (**b**) after 12 months (low ≤8 (n = 13); high >8 (n = 15)). SPPB groups were dichotomized with medians. Plots represent mean ± standard deviation.

PCA and PLS-DA of the urine spectrum for SPPB measured at baseline and 12 months later are presented in Figure 6. When comparing the groups at baseline (dichotomized by medians, higher scores denoting better performance), we observe a slight discrimination. When we compare the low and high total SPPB groups after 12 months, the separation remains similar. To understand these separations, we conducted a targeted analysis. As depicted in Figure 7a, we observed increased levels of the paracetamol derivatives, alanine, lactate, acetate, and acetone in the group with lower SPPB scores at baseline. After 12 months (Figure 7b), the group with lower scores presented higher levels of valine, lactate, acetone, hippuric acid, and TMAO at baseline when compared with the group with higher SPPB scores.

#### 2.3.4. Mortality over 24 Months

**Figure 8:**
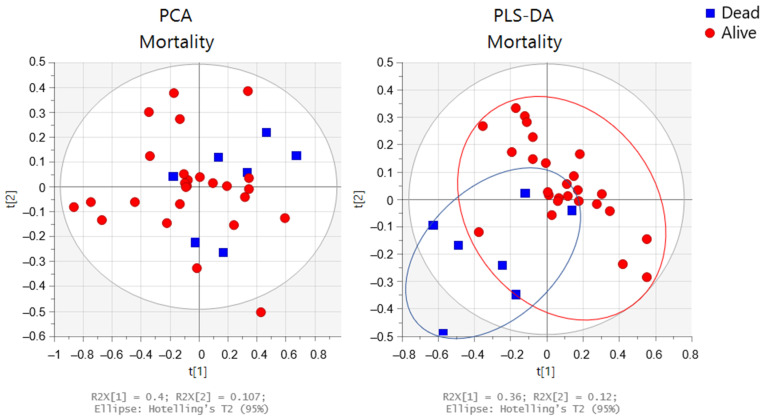
Multivariate analysis obtained from the baseline urine spectra from patients who remained alive (n = 26) (red circles) or died (n = 7) (blue squares) during the two-year study. Score plots from the PCA (**left** side) and PLS-DA (**right** side).

**Figure 9:**
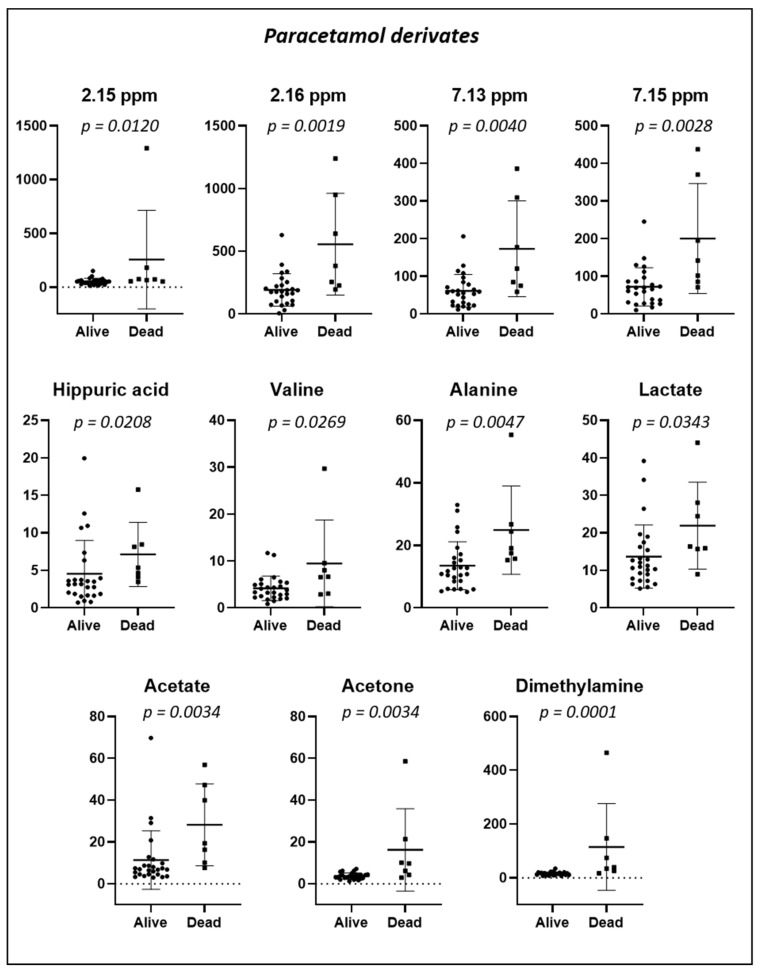
Dot plots of targeted metabolites showing significant differences in urinary concentration in specimens collected at baseline from 33 patients according to whether they were alive (n = 26) or died (n = 7) during the 24-month study period. Plots represent mean ± standard deviation.

The PCA and PLS-DA of the baseline urine spectra from patients during the two-year study (first randomized to exercise training or usual care; the second-year registry follow-up) are presented in Figure 8. When comparing spectra, we observed that the spectra of those patients who died were distributed on the lower left side of the PLS-DA score plot, whereas those of patients who were alive at 24 months were located on the other side, except for one spectrum. In addition, we conducted a targeted analysis. As shown in Figure 9, in patients who died, increased levels of eight urine metabolites (paracetamol derivates (2.15 ppm; 2.16 ppm; 7.13 ppm; and 7.15 ppm); hippuric acid; valine; alanine; lactate; acetate; acetone, and dimethylamine) were observed.

### 2.4. Pathway Analysis

**Figure 10:**
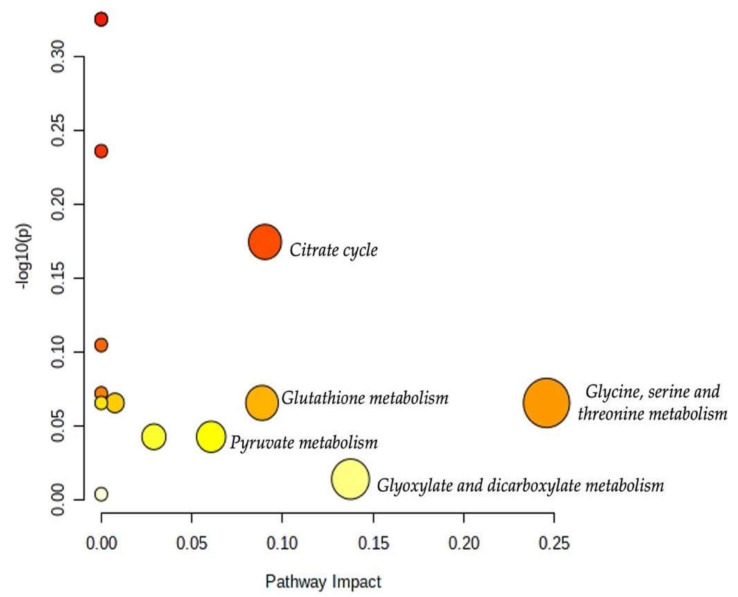
Overview of the pathway analysis arranged according to the scores from the enrichment analysis (y-axis) and the topology analysis (x-axis). The color and size of each circle (pathways) are based on its *p*-value and pathway impact value, respectively. Circles located in the top right diagonal region represent pathways with significant metabolite changes and higher impact. The pathway names with the highest impact values (largest circles) are mentioned (glycine, serine, and threonine metabolism; glyoxylate and dicarboxylate metabolism; citrate cycle; glutathione metabolism; pyruvate metabolism).

The results of the pathway analysis are presented in a graphical output (Figure 10) and in Table 3, which presents the matched metabolic pathways. The 19 identified urine metabolites are involved in 13 metabolic pathways.

As shown in Table 3, the topology analysis showed that seven pathways (glycine, serine, and threonine metabolism; glyoxylate and dicarboxylate metabolism; citrate cycle; glutathione metabolism; pyruvate metabolism; glycolysis/gluconeogenesis; primary bile acid biosynthesis) have a pathway impact. Among them, five pathways (glycine, serine, and threonine metabolism; glyoxylate and dicarboxylate metabolism; citrate cycle; glutathione metabolism; pyruvate metabolism) had an impact value higher than 0.05. However, the *p*-values of some of the enriched pathways were higher than 0.05 which may attenuate their impacts.

## 3. Discussion

In the present study, a ^1^H NMR-based metabolomic approach was used (1) to describe the urine metabolome in patients after hip fracture surgery, according to frailty severity (non-frail, pre-frail, and frail), functional independence (total, motor, and cognitive FIM) and physical performance (SPPB); (2) to explore the link between baseline metabolomic urinary profiles and physical performance (SPPB) after a one-year exercise program in a randomized trial, and (3) to investigate whether the urinary metabolite characteristics might predict patient survival (living or dead) over the 24-month study period. NMR-based metabolomics revealed major metabolic variations between groups with different profiles of frailty, FIM, and SPPB. These variations were quantified. The metabolic variations concerned ten identified urine metabolites: paracetamol derivatives (4 peaks: 2.15 ppm; 2.16 ppm; 7.13 ppm; and 7.15 ppm); hippuric acid; acetate; acetone; dimethylamine; glycine; alanine; lactate; valine; and TMAO.

Based on the frailty severity, our results revealed that the level of paracetamol derivatives in urine was significantly increased: (1) in frail persons (frailty score ≥ 3) in comparison to non-frail patients; (2) in persons with poor FIM scores (low level of total, motor, and cognitive independence) in comparison to persons with greater FIM scores; (3) in persons with low SPPB scores (poor physical performance) compared to persons with better FIM scores. As pain can be a major factor in worsening frailty, paracetamol is a model drug that is often used in the pain management in frail older adults [18,19,20,21,22] and also in pain relief after orthopedic surgery in older patients [23,24]. Paracetamol is metabolized in the liver and then excreted in the urine as a glucuronide or a sulfonate [18]. Aging induces a decline in the clearance of paracetamol in (frail) persons owing to decreased liver volume with age and altered activity of conjugating enzymes [18]. These issues can further explain the higher level of paracetamol derivatives in frail patients (among our patients, 23 used paracetamol orally at the time of discharge). Our results are in agreement with the observations of Koponen et al. [19], who noted that paracetamol use was more prevalent among frail compared to pre-frail or non-frail older people. Additionally, we observed an increased level of paracetamol in patients with impaired functional independence and/or with poor physical performance. Hip-fracture related pain compromises functional performance and affects activities of daily living [25], so the pain medication should be optimized to prevent pain complications. Our NMR analysis revealed that frail patients’ urine contained a higher level of paracetamol derivatives than that of non-frail patients.

Urine analysis also revealed an increased level of hippuric acid in frail patients compared with non-frail patients. Hippuric acid represents a glycine conjugate of benzoic acid [26]. Hippuric acid could be associated with hepatic function, renal clearance, and the degradation of certain dietary components [26,27]. Furthermore, it has been related to aging and geriatric syndromes in metabolomic studies [12,13,28]. Urinary hippuric excretion increases with aging [28], especially in frail people. This finding has been explained by many factors related to frailty, including age-related diseases, low fruit and vegetable intake, changes in the gut microflora, sarcopenia, inflammation, and hypomobility [10,25,26,28]. None of our patients had hippuric acid as medication, so the compound revealed in our investigation was certainly an endogenous metabolite.

Regarding FIM, urine analysis revealed significantly increased urine levels of paracetamol, acetate, acetone, dimethylamine, and glycine in patients who needed more assistance in daily activities. These metabolites, including ketone bodies, organic acids, and amino acids, are affected by the aging process and frailty and are related to impaired functional independence [29,30]. Acetone is a ketone body that is released by the liver from the adipose tissue and is excreted by the kidneys [31]. It acts as an energy source when glucose reserves are insufficient, especially during fasting. Ketone bodies increase in the case of hypoglycemia or when glucose molecules in the circulation cannot enter the cells. These substances can generate oxygen radicals and cause lipid peroxidation [32,33], which may play a role in the pathogenesis of vascular diseases and diabetes. Our analysis revealed that the excretion of ketone bodies increased in patients with low functional independence (FIM) compared to those with higher scores. This finding may be explained by glucose deficiency in these patients owing to a lower carbohydrate intake, energy metabolism imbalance, loss of appetite, and alterations in taste and smell [34,35]. Furthermore, frail people face a risk of malnutrition, which thereby contributes to a loss of muscle mass and thus a loss of muscle strength, which explains why frail patients present decreased motor FIM scores. We also observed that patients with lower FIM scores had increased acetate levels in their urine. Acetate is an anion formed by the removal of a hydrogen atom from acetic acid. It has been observed that oxidative stress phenomena are more present in older subjects with pathologies such as arthrosis and diminish in subjects who are more physically active [36]. Therefore, in patients with lower FIM who have difficulties being physically active, it is likely that a higher concentration of H_2_O_2_ (a common oxidative agent) could react with pyruvate to generate acetate (and CO_2_) via an oxidative decarboxylation reaction [37]. We also found an increased level of dimethylamine in patients with low FIM scores, with dimethylamine being the most abundant short-chain aliphatic amine in human urine. In older patients, a higher level of this urine metabolite has been associated with diseases related to renal and cardiovascular systems [38,39].

Regarding the SPPB, NMR-based analysis and univariate comparisons revealed that patients with poor physical performance (SPPB) vs. those with better SPPB at 12 months had higher levels of the following urine metabolites at baseline: paracetamol, acetate, acetone, hippuric acid, alanine, lactate, valine, and TMAO. Alanine, lactate, and valine are involved in antioxidant and anti-inflammatory responses [40,41,42,43]. A growing body of literature has reported that a chronic low-grade inflammatory state occurs in aging [44,45,46]. The immune system deteriorates progressively with age-related diseases [47], thereby contributing to increased circulatory inflammatory markers (TNF-alpha, IL-1beta, IL-6, COX-2, INOS) [47], impaired autophagy, redox imbalance, and increased oxidative stress [44,45]. These underlying mechanisms of aging (low-grade inflammation and oxidative stress) can influence the physical and functional ability of older adults, leading to decreased physical performance [48,49]. Accumulating evidence suggests that alanine and valine supplementation [40,41,42,43] could reduce inflammation and oxidative stress, and prevent decreased muscular strength and mass. However, our results revealed that patients with poor performance had increased urinary levels of alanine, valine, and lactate when compared to those with better performance. We can speculate that this difference could be linked to either (1) an increased β-alanine [50,51] and branched-chain amino acid supplementation [52,53] in more frail patients (however, our patients did not take supplements) or (2) a greater release of metabolites involved in the antioxidant and anti-inflammatory reactions. We also observed an increased urine level of trimethylamine N-oxide (TMAO), in patients with poor physical performance compared with those with better performance. An increased level of TMAO has often been linked to several metabolic and inflammatory disorders (obesity, higher risk of cardiovascular diseases) [54,55]. TMAO is considered to be an inflammatory mediator involved in the increased production of reactive oxygen species and the activation of the NLRP3 inflammasome in endothelial cells [56,57]. Our results demonstrated that patients with impaired physical performance had increased urine levels of metabolites involved in the systematic inflammatory response and oxidative stress (alanine, lactate, valine, and TMAO). These findings are in accordance with previous results [48,49] associating the decline in physical capacity with a state of chronic low-grade inflammation and oxidative stress that occurs with aging.

The urinary metabolite analysis taken at discharge from the rehabilitation hospital showed increased levels of paracetamol, lactate, acetate, acetone, valine, alanine, dimethylamine, and hippuric acid in patients who died during the two years following the hip fracture surgery. As discussed above, most of these urine metabolites are linked to metabolic, cardiovascular, renal, and inflammatory diseases. Our results confirmed previous findings reporting that the mortality risk increased with age-related disorders and diseases, including inflammation [58], oxidative stress [59], renal diseases [60], and malnutrition [61]. Our study should be considered a pilot study. Further metabolomic investigations are warranted to identify the metabolic profiles predisposing a higher risk of mortality.

The present study presents some limitations. The urinary samples of 33 successive patients comprise a subgroup of the randomized trial of 121 hip fracture patients. The specimen collection was difficult to organize in the hospital ward, many participants forgot that they were supposed to give a morning urinary sample, many participants were incontinent, had cognitive decline, and were unable to hold urine for the required time. However, our population is still representative of the initial population presented in the HIPFRA study [17] concerning age, frailty severity, and FIM and SPPB scores.

## 4. Materials and Methods

### 4.1. Study Design

This parallel design randomized clinical trial was conducted in one province (133,000 inhabitants) in Finland. The study received ethical approval from the Coordinating Ethics Committee of Helsinki University Hospital (Finland) in November 2014 and was registered at ClinicalTrials.gov (NCT02305433) in December 2014. The study was conducted in accordance with the Declaration of Helsinki, and all participants signed a written consent form. The study protocol [17], the results of the primary outcome [62], and of the secondary outcome functioning have been published [63].

### 4.2. Participants

The participants in this substudy participated in the larger HIPFRA study, for which patients with recently operated hip fractures were recruited from the rehabilitation hospital of South Karelia Social and Health Care District between December 2014 and December 2017. Patients were eligible for the study if they were at least 60 years old, living at home, able to walk indoors (walking aid allowed), had a Mini-Mental State Examination score of 12 or higher, and had a fracture that was the first operated proximal femoral fracture located in the femoral neck, pertrochanteric, or subtrochanteric region. Patients were excluded if they had contraindications to physical exercise, lived in a 24-h care facility, or had alcohol or drug abuse issues or severe illnesses with a life expectancy of less than two years. In addition, for the present subgroup study, a participant was eligible if he or she had given a urinary sample during his or her stay in the rehabilitation hospital between January 2015 and August 2017. Chronic diseases and prescribed drugs are presented in Supplementary Tables S1 and S2.

Eligible participants were randomized into two groups. The intervention group received a 12-month home-based physiotherapist-supervised training program twice a week, and the usual care group received health care services, including rehabilitation as needed [17].

### 4.3. Outcomes

The research physiotherapist or the research nurse performed the assessments of frailty status, Functional Independence Measure (FIM), and Short Physical Performance Battery (SPPB) at the participant’s home at baseline and after 12 months. The severity of frailty was assessed using Fried et al.’s (2001) [1] frailty phenotype classification (slightly modified), which contained five domains: exhaustion, slowness, weight loss, weakness, and physical activity. The Functional Independence Measure (FIM) [64] includes 18 tasks, of which 13 are motor (e.g., eating, toileting) and five are cognition tasks (e.g., problem solving and memory). The assessor evaluated the participant’s capability to perform the tasks by interviewing and observing the participant during the home visits. The tasks were graded using a scale from 7 (completely independent) to 1 (needs total assistance) with a maximum total score of 126. The Short Physical Performance Battery (SPPB) measures physical performance and includes three tests: a balance test, a 4-m walk, and a chair rise test, each of which are scored from 0 to 4, with a maximum total score of 12 points. Mortality over 24 months was acquired from the medical records.

### 4.4. Urine Sample Collection

Urine samples were collected in the rehabilitation hospital ward from voluntary patients who were potentially eligible for the HIPFRA study. For the dinner preceding the morning urine collection, the patients were advised to abstain from fish and other seafood. Morning urine samples were collected a few days before discharge. The patient needed to be able to hold urine for at least 4 h before voiding into a collection cup. The sample was taken from the first urination in the morning. The research nurse pipetted a 10-mL sample from the collection bowl, and the pipetted sample was stored in the tube without preservatives. The sample tubes were immediately frozen and preserved in a freezer at −80 °C. A urine sample could not be taken if the patient was incontinent or had a low cognitive status. After all the samples were collected, they were sent to Poitiers, France, in a dry ice container for analysis. Thirty-three samples were analyzed with NMR spectroscopy.

### 4.5. Sample Preparation for NMR Analysis

Urine samples were thawed at room temperature and homogenized with vortexing. A total of 400 µL of urine was mixed with 200 µL of phosphate buffer prepared in deuterium oxide containing 0.05 wt. % 3-(trimethylsilyl) propionic-2,2,3,3-d4 acid, sodium salt (TSP). Samples were vortexed for 15 s and allowed to stand for 15 min at room temperature. Samples were vortexed for 15 s and centrifuged at 8000 *g* at room temperature for 5 min. The pH of the supernatant was adjusted to 7.40 ± 0.05 with the addition of concentrated chlorhydric acid or sodium hydroxide. Five hundred microliters were introduced into a 5 mm Pyrex NMR sample tube. NMR analysis was performed immediately after the preparation [65]. Solvents were purchased from Sigma Aldrich^®^ (Darmstadt, Germany), and NMR tubes were purchased from CortecNet^®^ (Les Ulis, France), Proton NMR Spectroscopy. All NMR experiments and data analyses were performed randomly over one unique period.

### 4.6. Instrument Description

Spectra were obtained with a 500 MHz SB Bruker Avance Neo spectrometer at 11.75 Tesla (BioSpin^®^, Wissenbourg, France). The magnet was equipped with a 5 mm BBO cryoprobe prodigy (Bruker BioSpin^®^, Wissebourg, France). Tuning and matching were automated. Automated gradient shimming for Z coils was used and manual optimization was performed if needed. The spectrometer was controlled with TopSpin 4.0 software (Bruker BioSpin^®^, Wissenbourg, France).

### 4.7. Spectrum Acquisition Parameters

Samples were thermostated at 293 K without spinning. One-dimensional (1-D) ^1^H NMR spectra were obtained at 500.09 MHz using a 1-D experiment impulsion acquisition sequence with a noesygppr1d pulse sequence with a presaturation delay during the relaxation delay (d1 = 5 s) and mixing time (d8 = 10 ms). The homospoil/gradient pulse p16 was 1 ms and the delay for homospoil/gradient recovery was 200 µs. Spectra were obtained in 12 min and 15 s by accumulating 64 free induction decay (FID) and 4 dummy scans. The acquisition time was 5.57 s with a spectral width of 6 kHz collected at 64 K data points. The 90° pulse delay was 12 µs with a power level of −12.14 dB. In addition, the residual water resonance was presaturated with a 40.26 dB field strength irradiation. The receiver gain was set at 32 for each sample.

### 4.8. Spectrum Processing Parameters

All spectra were processed with TopSpin 4.0 software (Bruker BioSpin^®^, Wissenbourg, France). Typical processing parameters were the application of a Fourier transform without line broadening or zero filling. Chemical shifts (δ in ppm) were reported relative to the signal of TSP at 0 ppm. Phase correction and baseline correction were performed manually. The relative values obtained for each metabolite were obtained by integrating the signal relative to the TSP signal.

Identification of the metabolites was performed using the Human Metabolome Database [66], the Magnetic Resonance Metabolomics Database [67], our database performed at pH = 7.4, and literature on urinary NMR analysis [68,69]. According to the Metabolite Identification Task Group from the Metabolomics Standards Initiative, the level of identification was 2 [70]. Raw data were registered in the MetaboLights database [71] (MTBLS2903).

### 4.9. Statistical Analysis

For frailty severity, the population was classified as non-frail (none of the five criteria present, i.e., robust; 5 persons), pre-frail (1–2 criteria; 18 persons), or frail (3 or more criteria; 10 persons) [72]. For other outcomes (age, SPPB, and FIM), we used their medians to create the groups.

#### 4.9.1. Multivariate Analysis

We performed automatic bucketing with a bucket width of 0.05 ppm, normalized to the total integral (from 0.6 to 10 ppm with the exclusion of water signal and urea from 4.5 to 6.5 ppm). We used the Amix viewer from Bruker^®^ to generate matrices. We used Pareto scaling before generating the score and loading plots from PCA and PLS-DA using Simca^®^ 17 software from Sartorius AG (Goettingen, Germany). We also generated contribution plots to help find the most discriminative variables.

#### 4.9.2. Univariate Analysis

Statistical analyses were performed using the statistical software Prism 8 (GraphPad software© (San Diego, CA 92108, US). The Gaussian distribution was tested for each variable using the Shapiro–Wilk test. A paired t-test or nonparametric Wilcoxon test was used to assess differences between metabolite concentrations in urines according to the parameters of interest. The results of the tests were considered significant at *p* ≤ 0.05. The results were expressed as means with the standard deviations (SD) or with boxplots.

#### 4.9.3. Pathway Analysis

We performed a pathway analysis using MetaboAnalyst 5.0 on 20 July 2021 (https://www.metaboanalyst.ca), an easy-to-use web-based tool suited to comprehensive and integrative metabolomics data analysis [73]. The metabolite table was autoscaled. We used the KEGG (Kyoto Encyclopedia of Genes and Genomes) library for Homo sapiens and we have launched two forms of pathway analysis, enrichment (global test) and topology (relative-betweenness centrality), to assess the *p*-values and the pathway impact values, respectively.

## 5. Conclusions

Our preliminary findings with the NMR-based metabonomic approach reveal that among patients after hip fracture surgery, frail patients, and those patients with impaired functional independence or poor physical performance, showed increased levels of urine metabolites associated with metabolic, inflammatory, and renal disorders related to aging, such as a low-grade inflammation, oxidative stress, and other age-related problems (e.g., malnutrition, low physical activity). More metabolomic studies with other biological fluids (plasma) and larger populations are warranted to identify metabolic factors predisposing older patients to frailty and impaired performance. The NMR-based metabonomic approach could be a tool of importance in comparing the metabolic profiles of older adults, e.g., after major surgery when planning rehabilitation.

## Figures and Tables

**Table 1:** Baseline characteristics of the participants (all and by their frailty status). Results are expressed as means ± SD.

Variables				
	All Patients; n = 33	Non-Frail; n = 5	Pre-Frail; n = 18	Frail; n = 10
Age (years)	80.0 ± 8.0	79.4 ± 8.3	79.5 ± 8.0	81.0 ± 7.0
BMI	25.3 ± 3.7	25.2 ± 3.1	26.1 ± 3.7	23.8 ± 4.0
Frailty score	2.0 ± 1.4	0.0	1.6 ± 0.5 *	3.8 ± 0.8 ^†,^*
SPPB score	3.7 ± 2.0	5.2 ± 2.8	3.8 ± 1.9	2.9 ± 1.1
Total FIM score	101.4 ± 11.5	108.4 ± 8.9	103.1 ± 10.5	95.0 ± 12.3
Motor FIM score	71.1 ± 8.8	76.0 ± 7.6	72.8 ± 6.8	65.6 ± 10.5
Cognitive FIM score	30.3 ± 4.3	32.4 ± 3.0	30.2 ± 3.0	29.3 ± 3.5

* *p* < 0.05 vs. non-frail. ^†^ *p* < 0.05 vs. pre-frail.

**Table 2:** NMR chemical shift (δ) and Human Metabolome Database identification number (HMDB ID) for selected metabolites.

Number	Metabolites	HMDB ID	δ ^1^H in ppm (Multiplicity)
1	L-Valine	HMDB0000883	0.99 (d)
2	L-Lactic acid	HMDB0000190	1.33 (d)
3	L-Alanine	HMDB0000161	1.48 (d)
4	Acetic acid	HMDB0000042	1.92 (s)
5	Paracetamol (Acetaminophen)	HMDB0001859	2.15 (s)
6	Acetaminophen glucuronide	HMDB0010316	2.17 (s)
7	Acetaminophen sulfate	HMDB0059911	2.18 (s)
8	Acetone	HMDB0001659	2.24 (s)
9	Citric acid	HMDB0000094	2.55 and 2.69 (syst AB)
10	Dimethylamine	HMDB0000087	2.72 (s)
11	Methylguanidine	HMDB0001522	2.83 (s)
12	Creatinine	HMDB0000562	3.05 (s)
13	Dimethyl sulfone	HMDB0004983	3.15 (s)
14	Trimethylamine N-oxide	HMDB0000925	3.27 (s)
15	Glycine	HMDB0000123	3.57 (s)
16	Urea	HMDB0000294	5.8 (broad)
17	Acetaminophen derivates	HMDB0001859	7.13 and 7.15 (syst AB)
		HMDB0010316	
		HMDB0059911	
18	Hippuric acid	HMDB0010316	7.65 (t)
19	Formic acid	HMDB0059911	8.46 (s)

**Table 3:** Results of pathway analysis with MetaboAnalyst 5.0 (https://www.metaboanalyst.ca) indicating the total number of involved metabolites and those detected in our data based on the *p*-value and the impact of each metabolic pathway. Analysis performed on 20 July 2021.

	Metabolites		Pathway Analysis	
	Total Number	Detected	*p*-Value	Impact
Glycine, serine, and threonine metabolism	33	1	0.861	0.25
Glyoxylate and dicarboxylate metabolism	32	4	0.969	0.14
Citrate cycle (TCA cycle)	20	1	0.669	0.09
Glutathione metabolism	28	1	0.861	0.09
Pyruvate metabolism	22	2	0.907	0.06
Glycolysis/Gluconeogenesis	26	2	0.907	0.03
Primary bile acid biosynthesis	46	1	0.861	0.001

## Data Availability

Data supporting reported results are stored at the Faculty of Medicine, University of Poitiers.

## Supplementary Materials

The following are available online at https://www.mdpi.com/article/10.3390/metabo12080744/s1, Figure S1. Overall PCA for all urine samples (n = 33). (a) Score plot; (b) loading plot and (c) contribution plot (one outlier vs other’s spectra); Figure S2. Dot plots of targeted metabolites that were significantly different between patients younger than 80 years old (Group 1, n = 17) or older than 80 years old (Group 2, n = 16); Figure S3. Dot plots of targeted metabolites that were significantly different between women (n = 26) and men (n = 7); Table S1. Drugs used at the time of the urine collection in the rehabilitation hospital, according to ATC (Anatomical Therapeutic Chemical Classification) codes. Analgesics are bolded (N02A opioids; N02B other analgesics and antipyretics. e.g., paracetamol). Each line corresponds to one subject; Table S2. Chronic diseases among all participants and according to frailty status. Data are expressed as number of patients and frequency (%). Table S3: VIP (Variable Importance in Projection) scores.

## Abbreviations

BMI	Body Mass Index (weight/height^2^)
COX	Cyclooxygenase
FIM	Functional Independence Measure
HMDB	Human Metabolome Database
INOS	Inducible Nitric oxide synthase
KEGG	Kyoto Encyclopedia of Genes and Genomes
NLRP3	NLR Family Pyrin Domain Containing 3
NMR	Nuclear Magnetic Resonance
PC	Principal Component
PCA	Principal Component Analysis
PLS-DA	Partial Least Square—Discriminant Analysis
SPPB	Short Physical Performance Battery
TMAO	Trimethylamine N-oxide
TSP	3-(trimethylsilyl)propionic-2,2,3,3-d4 acid, sodium salt
δ	Chemical shift
