# Supplementary material for: 1H NMR Urinary Metabolomic Analysis in Older Adults after Hip Fracture Surgery May Provide Valuable Information for Patient Profiling—A Preliminary Investigation
Source: Metabolites. 2022 Aug 12;12(8):744. doi: 10.3390/metabo12080744 (PMC9415398; doi:10.3390/metabo12080744)
Supplement: Supplementary file 1 [file metabolites-12-00744-s001.zip › metabolites-1809646-supplementary.pdf]

Figure S1. Overall PCA for all urine samples (a) score plot; (b) loading plot and (c) contribution plot

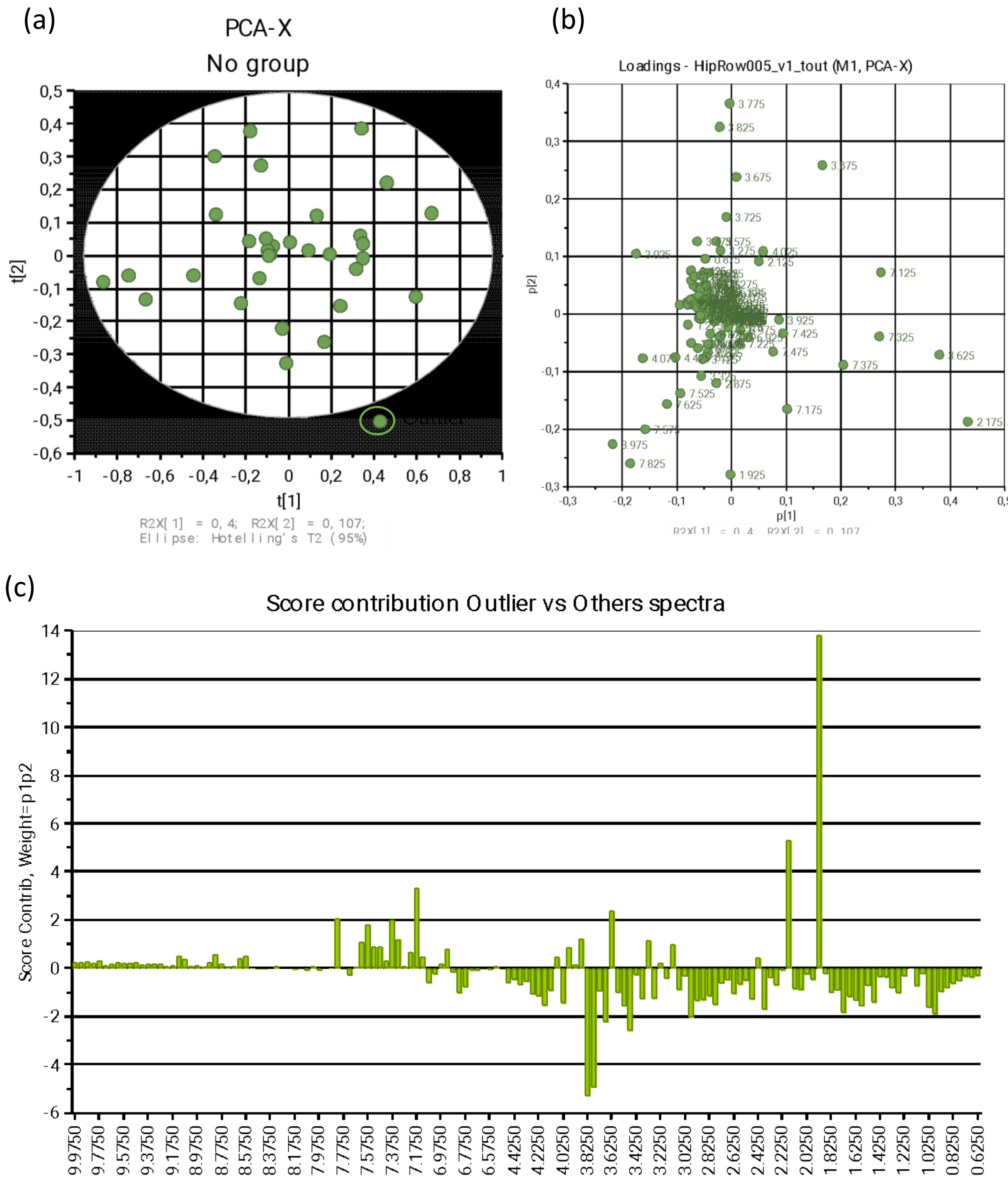

Figure S2. Dotplots of targeted metabolites that were significantly different between patients aged younger than 80 years (Group 1; n=17) or older than 80 years (Group 2; n=16). Plots represent mean ± standard deviation.

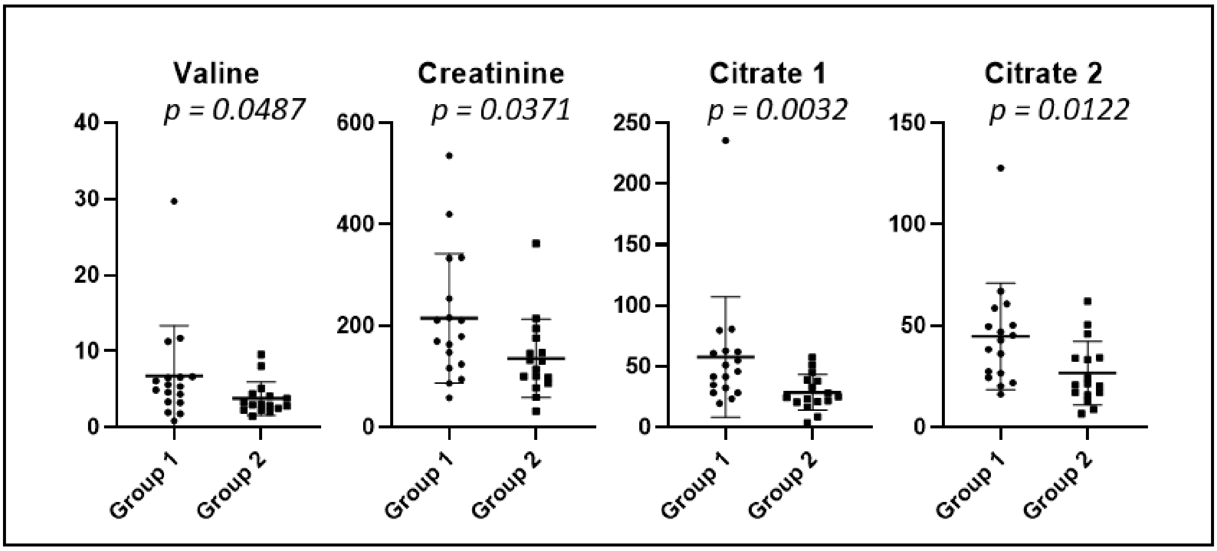

Figure S3. Dotplots of targeted metabolites that were significantly different between women (n=26) and men (n=7). Plots represent mean ± standard deviation.

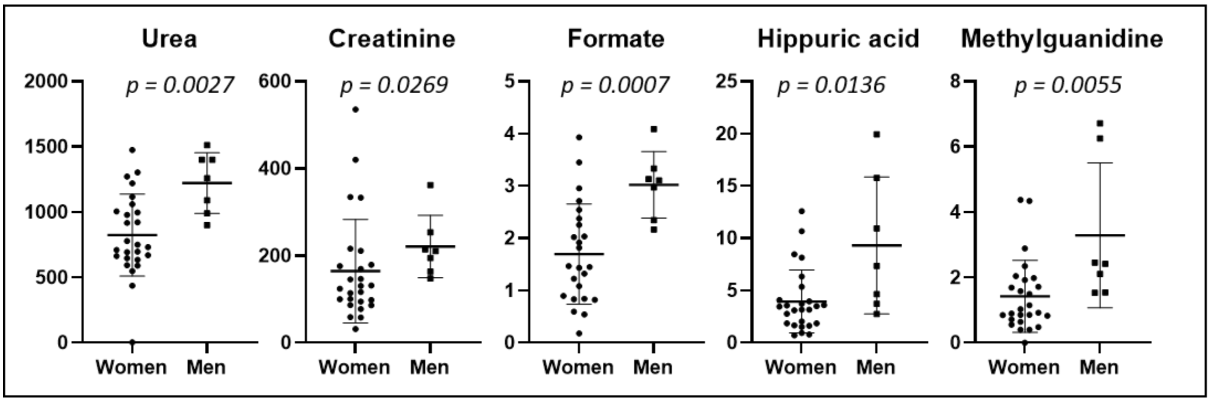

Table S1. List of ATC (Anatomical Therapeutic Chemical Classification) codes of drugs used at the time of the urine collection in the rehabilitation hospital. Analgesics are highlighted. Each line corresponds to a subject.

A06AD11	B01AA03	M02AC										
A02BC02	A03FA01	A04AA01	A12AX	B01AB05	B03AA01	H02AB02	nutricial drink	**N02AA05**	**N02AA05**	**N02AA55**	**N02BE01**	
A12AX	J01MA12	R05CB15	R05FA02									
A06AD65	A12AX	C01DA02	D07BA04	**N02AJ06**	N05CH01	S01XA20	B03AA01	M05BA08	**N02BE01**			
A02BC02	A12AX	B01AB05	B03AA01	C09CA01	M01AE01	M01AE03	**N02AA05**	**N02AA05**	**N02AA55**	**N02BE01**	**N02BE01**	
B01AB05	C08CA01	J01DB01	J01DC02	**N02AA05**	**N02AA05**	N02AE01	**N02BE01**					
A10AD05	B01AB05	C03CA01	C03DA01	J01DB01	J01DC02	**N02AA05**	**N02AA55**	**N02BE01**	**N02BE01**	N05AH04	N05AH04	
B01AB05	B03BB01	G04BD12	H02AB06	**N02AA05**	**N02AA55**	N02AE01	**N02BE01**	R02AB30	R05CB01	R05CB15	energy drink	
B01AB05	A12AX	B03AA01	G04CB02	J01DC02	**N02AA05**	**N02AA55**	**N02BE01**	N05CH01				
A02BC03	A10AB05	A10AE04	A10BA02	A12AA04	B01AB05	C01DA08	C08CA01	C09CA06	**N02AA05**	**N02BE01**		
A02BC02	A10AB05	A12AX	B03AA01	C07AB07	H02AB06	**N02AA05**	**N02AA55**	**N02BE01**	R03AC02	R03AK07		
A04AA02	multivitamin	A12AX	B01AB05	C07AB02	J01XE01	M05BA08	**N02AA05**	**N02BE01**				
A04AA01	multivitamin	B01AA03	H02AB09	G04BD12	J01EA01	M05BA08	N02AA05	**N02AA05**	**N02BE01**			
A04AA02	H02AB02	J05AB11	**N02AA05**	N05AD08	**N02BE01**							
A02BC02	A06AD65	A12AX	B01AA03	C03CA01	C07AB07	C09CA01	M05BA08	C10AA07	**N02AA55**	**N02AA05**		
A02BC02	A03FA01	multivitamin	A12AX	B01AB05	B03AA01	M05BA08	**N02AA05**	**N02AA55**	**N02BE01**			
A02BA02	A06AC01	A04AA01	A06AD65	B01AA03	B01AB05	C10AA05		H02AB02	C03CA01	M05BA08	**N02AA05**	
A03DA02	A04AA02	A10AB05	A10AE05	A10BA02	A10BH01	d3 vitamin	multivitamin	A12AX	B01AB05	B03AA01	C09AA04	
B01AA03	A04AA02	A12AX	B03AA01	C07AB02	C08CA01	C09CA01	M05BA08	N02AA05	**N02AA55**	**N02BE01**		
A06AD65	A12AX	B01AA03	B03AA07	C07AB07	C09CA01	G04CB02	multivitamin	J01CR02	**N02AA05**	**N02AA55**	**N02BE01**	
M05BA04	A12AX	**N02AA55**	N06AX11	N05CD07								
A02BC05	A06AD11	multivitamin	A12AX	B01AC30	B03AA01	B03BA03	C01DA08	C07AB02	C08CA01	M05BA08	**N02BE01**	
**N02AA55**	M05BA08	N06AX11	N05AD08	**N02BE01**	**N02AA55**	**N02AA05**	H02AB02	B03AA01	A12AX	A04AA02		
**N02AA55**	**N02AA05**											
A06AD65	multivitamin	A12AX	B01AF01	B03AA01	C07AB07	G03CA03	M05BA08	**N02AA05**	**N02AA55**	N05CH01		
B01AB05												
B01AB05	B01AC06	C03DA01	C07AB07	CA09AA05	C10AA05	M05BA04	M05BX04	**N02AA05**	**N02AA55**		**N02BE01**	
A12BA01	J01CA08	J01EA01	**N02BE01**	N05BA01	N05BA01	N05CF01						
A06AB08	A06AD65	multivitamin	A12AX	B01AB05	B02AA02	C03CA01	J01DC02	M05BA04	**N02AA05**	**N02AA55**	**N02BE01**	
multivitamin	B01AB05	N05BA01	M01AB05	A11DA01	A04AA01							
A06AD11	A06AD15	lactobacillus GG	multivitamin	A12AA04	B01AA03	A12BA01	B03BA03	J01DC02	**N02AA05**	**N02AA55**	**N02BE01**	
B01AB05	B03BB01	L04AX03	oxynorm									
A02BC02	multivitamin	A12AA04	A12BA01	B01AF01	multivitamin	C01AA05	C01DA08	C07AB07	**N02AA05**	**N02AA55**	**N02BE01**	

Table S2 . List of diseases among all participants and according to frailty status. Data are expressed as number of patients and frequency (%).

Diseases (frequency (%))	**All (n=33)**	**non-frail (n=5)**	**pre-fail (n=18)**	**frail (n=10)**
Hypertension	23 (70)	2 (40)	14 (78)	7 (70)
Diabetes	7 (21)	1 (20)	4 (22)	2 (20)
Cardiovascular diseases*	31 (94)	5 (100)	17 (94)	9 (90)
Musculoskeletal diseases**	29 (88)	5 (100)	17 (94)	7 (70)
Chronic urinary infection or other renal decease	4 (12)	0 (0)	2 (11)	2 (20)
Problems with voiding e.g.. incontinence	17 (52)	1 (20)	12 (67)	4 (40)
Osteoporosis	7 (21)	0 (0)	4 (22)	3 (30)

*including coronary hearth disease. angina pectoris. history of myocardial infarction. heart failure; **Musculoskeletal including arthritis. rheumatoid arthritis.

Table S3. Supplementary documents concerning the Variable Importance in Projection (VIP) score for each outcome.

VIP for PLS-DA regarding mortality

Primary ID (chemical shift)	VIP	Targeted metabolites
3.27500033" "	3.876950026	
**2.17500019" "**	**3.560869932**	Paracetamol (Acetaminophen) + Acetaminophen glucuronide
**7.12500048" "**	**2.995959997**	Acetaminophen derivates
4.07500029" "	2.932940006	
3.62500024" "	2.917439938	
2.87500024" "	2.859430075	
**7.17500019" "**	**2.733180046**	Acetaminophen derivates
4.02500010" "	2.414190054	
7.37500048" "	2.206650019	
7.32500029" "	2.185909986	
3.97500038" "	2.166569948	
3.02500033" "	2.10048008	
**1.92500031" "**	**1.85157001**	Acetate
7.82500029" "	1.733360052	
2.12500024" "	1.541049957	
7.57500029" "	1.536720037	
3.87500024" "	1.351250052	
3.22500038" "	1.332299948	
7.52500057" "	1.330680013	
**2.72500038" "**	**1.204300046**	Dimethylamine
**7.62500048" "**	**1.198559999**	Hippuric acid
1.87500024" "	1.162320018	
3.57500029" "	1.149960041	
**2.22500038" "**	**1.14151001**	Acetone
4.42500019" "	1.111529946	
3.77500033" "	1.072299957	
3.72500038" "	1.05456996	
3.07500029" "	0.96401298	
3.17500019" "	0.955281973	
8.22500038" "	0.909794986	
2.67500019" "	0.898232996	
1.22500026" "	0.893936992	
1.17500019" "	0.809105992	
3.92500019" "	0.777442992	
1.67500031" "	0.766892016	
3.12500024" "	0.766375005	

2.47500038" "	0.759234011	
7.42500019" "	0.743125975	
3.82500029" "	0.738216996	
8.82500076" "	0.727976978	
2.27500033" "	0.722187996	
2.57500029" "	0.71172303	
2.52500033" "	0.702306986	
**1.32500029" "**	**0.698172987**	Lactate
7.47500038" "	0.687752008	
1.77500021" "	0.685276985	
3.32500029" "	0.678472996	
3.37500024" "	0.677133024	
4.12500048" "	0.675014973	
1.42500031" "	0.674727023	
2.37500024" "	0.664951026	
3.52500033" "	0.663039029	
1.37500024" "	0.641982019	
**1.47500026" "**	**0.635479987**	Alanine
2.42500019" "	0.625910997	
3.42500019" "	0.62570399	
2.07500029" "	0.619328022	
1.72500026" "	0.609368026	
2.62500024" "	0.588383019	
8.07500076" "	0.573998988	
8.57500076" "	0.57209003	
2.32500029" "	0.563531995	
1.97500026" "	0.556529999	
7.07500029" "	0.556261003	
4.17500019" "	0.549516976	
9.12500000" "	0.549178004	
**7.67500019" "**	**0.529605985**	Hippuric acid
4.47500038" "	0.529430985	
2.02500033" "	0.512973011	
1.12500024" "	0.504921973	
3.47500038" "	0.497173011	
1.82500029" "	0.493827999	
**0.97500026" "**	**0.493310004**	Valine
6.82500029" "	0.483013004	
6.87500048" "	0.479968011	
1.62500024" "	0.470990986	
1.52500021" "	0.470589012	
0.92500025" "	0.469552994	
1.57500029" "	0.467061996	

1.27500021" "	0.464507997	
6.97500038" "	0.460750997	
1.07500029" "	0.450549006	
7.87500048" "	0.448967993	
2.82500029" "	0.446545005	
2.92500019" "	0.440290987	
2.77500033" "	0.436857015	
4.37500048" "	0.429668009	
6.62500048" "	0.42809099	
4.22500038" "	0.427345991	
7.22500038" "	0.421555996	
8.27500057" "	0.421526998	
4.32500029" "	0.410066009	
8.12500000" "	0.408854991	
0.87500024" "	0.405831993	
7.97500038" "	0.39586699	
2.97500038" "	0.379444987	
4.27500010" "	0.37340501	
8.32500076" "	0.365776986	
8.17500019" "	0.344644994	
8.87500000" "	0.343387991	
8.62500000" "	0.339143008	
0.82500023" "	0.327154994	
1.02500021" "	0.323163986	
0.77500021" "	0.314696997	
7.92500019" "	0.312976003	
0.72500026" "	0.310793996	
7.72500038" "	0.303645015	
7.27500057" "	0.302733004	
6.67500019" "	0.291162997	
8.37500000" "	0.286697	
7.77500057" "	0.283562988	
6.77500057" "	0.275002003	
7.02500057" "	0.270597011	
8.47500038" "	0.263000011	
6.92500019" "	0.262486994	
6.72500038" "	0.245100006	
3.67500019" "	0.233806998	
0.62500024"	0.228296995	
8.52500057" "	0.228003994	
0.67500025" "	0.215964004	
6.57500029" "	0.200363994	
8.42500019" "	0.175882995	
6.52500010" "	0.157995999	
8.02500057" "	0.104621999	
8.67500019" "	0.085220799	

9.07500076" "	0.082114503	
9.02500057" "	0.079643801	
9.92500019" "	0.073346503	
8.97500038" "	0.068037197	
9.97500038" "	0.065927498	
9.82500076" "	0.065760799	
9.87500000" "	0.064868197	
8.77500057" "	0.064815402	
9.52500057" "	0.063196503	
9.37500000" "	0.063153602	
9.62500000" "	0.060855899	
9.42500019" "	0.059576701	
9.17500019" "	0.058416001	
9.27500057" "	0.057606298	
9.77500057" "	0.056871999	
9.47500038" "	0.056492001	
9.32500076" "	0.055498101	
9.67500019" "	0.052842502	
9.57500076" "	0.051442001	
9.72500038" "	0.0470392	
8.92500019" "	0.044805601	
9.22500038" "	0.0435877	
8.72500038" "	0.042292599	
4.52500010" "	0.031485599	

VIP for PLS-DA regarding non-frail vs. pre-frail vs. frail

Primary ID (chemical shift)	VIP	Targeted metabolites
**2.17500019" "**	**2.621119976**	Paracetamol (Acetaminophen) + Acetaminophen glucuronide
3.02500033" "	2.617899895	
3.62500024" "	2.466789961	
3.87500024" "	2.466470003	
**7.12500048" "**	**2.291450024**	Acetaminophen derivates
**7.17500019" "**	**2.124409914**	Acetaminophen derivates
3.77500033" "	2.122129917	
2.12500024" "	2.034060001	
4.07500029" "	1.972609997	
3.17500019" "	1.931540012	
7.42500019" "	1.870519996	
7.52500057" "	1.812280059	

7.82500029" "	1.762500048	
2.32500029" "	1.721240044	
2.27500033" "	1.69216001	
7.32500029" "	1.684129953	
3.82500029" "	1.653949976	
4.02500010" "	1.633700013	
4.17500019" "	1.543280005	
3.97500038" "	1.519109964	
2.07500029" "	1.480399966	
3.67500019" "	1.415519953	
3.22500038" "	1.413550019	
3.52500033" "	1.365589976	
2.47500038" "	1.322620034	
7.57500029" "	1.312250018	
1.42500031" "	1.270120025	
1.97500026" "	1.263640046	
1.77500021" "	1.254109979	
3.12500024" "	1.239650011	
3.27500033" "	1.233690023	
7.62500048" "	1.213289976	
1.72500026" "	1.203529954	
4.32500029" "	1.20144999	
2.87500024" "	1.196760058	
4.22500038" "	1.196689963	
2.67500019" "	1.158910036	
1.62500024" "	1.158480048	
7.47500038" "	1.132200003	
3.57500029" "	1.110460043	
3.47500038" "	1.09691	
2.02500033" "	1.095469952	
7.37500048" "	1.086770058	
3.07500029" "	1.083220005	
1.87500024" "	1.079749942	
2.72500038" "	1.076120019	
4.27500010" "	1.041120052	
1.57500029" "	1.000499964	
2.42500019" "	0.994534016	
4.42500019" "	0.985616028	
3.92500019" "	0.984224021	
4.37500048" "	0.971475005	
0.87500024" "	0.96302402	
2.22500038" "	0.957993984	
4.47500038" "	0.943037987	
1.82500029" "	0.940477014	
7.92500019" "	0.920060992	
2.52500033" "	0.900605023	

2.92500019" "	0.891274989	
1.67500031" "	0.871165991	
8.82500076" "	0.839631975	
7.22500038" "	0.832316995	
2.57500029" "	0.825942993	
1.07500029" "	0.81397599	
4.12500048" "	0.807277024	
2.77500033" "	0.803331971	
2.37500024" "	0.796149015	
2.97500038" "	0.778917015	
1.17500019" "	0.750138998	
1.47500026" "	0.717920005	
1.92500031" "	0.710305989	
8.57500076" "	0.686053991	
0.72500026" "	0.650497019	
7.67500019" "	0.63865602	
2.62500024" "	0.614539981	
9.12500000" "	0.609982014	
7.02500057" "	0.59641403	
1.22500026" "	0.588941991	
8.47500038" "	0.572656989	
3.37500024" "	0.56212002	
7.27500057" "	0.560696006	
6.97500038" "	0.559583008	
6.92500019" "	0.559319019	
2.82500029" "	0.539941013	
0.97500026" "	0.530727983	
3.42500019" "	0.511327028	
7.77500057" "	0.508264005	
0.92500025" "	0.491261989	
8.62500000" "	0.471403003	
1.37500024" "	0.465869009	
6.62500048" "	0.463712007	
3.72500038" "	0.462334007	
7.07500029" "	0.46154201	
3.32500029" "	0.429926008	
1.32500029" "	0.42850399	
6.82500029" "	0.421849996	
0.67500025" "	0.420361996	
1.27500021" "	0.384627014	
0.82500023" "	0.381965011	
6.57500029" "	0.381561995	
1.02500021" "	0.375689	
1.52500021" "	0.368059993	
6.67500019" "	0.364618003	
1.12500024" "	0.360635996	

7.72500038" "	0.33949101	
8.87500000" "	0.329645008	
0.77500021" "	0.315535009	
8.07500076" "	0.309215993	
6.72500038" "	0.304587007	
0.62500024"	0.290042996	
6.52500010" "	0.248482004	
8.17500019" "	0.236695006	
8.32500076" "	0.224267006	
8.22500038" "	0.195954993	
8.27500057" "	0.191910997	
8.67500019" "	0.183440998	
7.97500038" "	0.177037999	
8.52500057" "	0.169108003	
9.07500076" "	0.168966994	
8.42500019" "	0.166018993	
8.37500000" "	0.163282007	
6.87500048" "	0.160127997	
6.77500057" "	0.154459998	
7.87500048" "	0.139191002	
8.12500000" "	0.13651	
8.77500057" "	0.136365995	
9.97500038" "	0.131915003	
9.02500057" "	0.130785003	
8.02500057" "	0.129278004	
9.92500019" "	0.129040003	
9.87500000" "	0.127765998	
9.82500076" "	0.127290994	
9.62500000" "	0.125210002	
9.17500019" "	0.124076001	
9.37500000" "	0.123880997	
8.72500038" "	0.123186	
9.67500019" "	0.12314	
9.52500057" "	0.121652	
9.32500076" "	0.121462002	
8.92500019" "	0.120945998	
9.47500038" "	0.119561002	
9.42500019" "	0.118308999	
9.77500057" "	0.116705999	
9.57500076" "	0.113314003	
9.22500038" "	0.112209998	
8.97500038" "	0.109160997	
9.27500057" "	0.095765002	
4.52500010" "	0.092202596	
9.72500038" "	0.087254703	

VIP for PLS-DA regarding non-frail vs. frail		
Primary ID (chemical shift)	VIP	Targeted metabolites
3.62500024" "	3.454440117	
3.02500033" "	3.097249985	
**2.17500019" "**	**2.919209957**	Paracetamol (Acetaminophen) + Acetaminophen glucuronide
**7.12500048" "**	**2.666670084**	Acetaminophen derivates
7.32500029" "	2.505949974	
3.57500029" "	2.278759956	
4.07500029" "	2.217700005	
3.87500024" "	2.185780048	
7.42500019" "	2.125240088	
3.27500033" "	2.102649927	
3.97500038" "	1.812489986	
4.42500019" "	1.651720047	
3.92500019" "	1.627429962	
2.87500024" "	1.615700006	
7.37500048" "	1.57573998	
7.82500029" "	1.514790058	
4.02500010" "	1.505769968	
7.57500029" "	1.479060054	
7.27500057" "	1.459339976	
3.52500033" "	1.458119988	
3.67500019" "	1.395799994	
7.47500038" "	1.374060035	
7.22500038" "	1.368549943	
3.72500038" "	1.365759969	
3.22500038" "	1.340330005	
4.32500029" "	1.295719981	
1.87500024" "	1.27128005	
2.32500029" "	1.167850018	
1.92500031" "	1.150570035	
8.82500076" "	1.106809974	
2.12500024" "	1.083619952	
0.92500025" "	1.076050043	
1.47500026" "	1.071099997	
1.32500029" "	1.041120052	
1.42500031" "	1.038969994	
7.02500057" "	1.037529945	
3.07500029" "	1.021450043	
2.47500038" "	1.013219953	

1.17500019" "	0.998085976	
1.52500021" "	0.995872021	
**7.62500048" "**	**0.972616971**	Hippuric acid
1.57500029" "	0.964936018	
3.12500024" "	0.945870996	
2.27500033" "	0.940083981	
2.67500019" "	0.920063019	
2.57500029" "	0.910143971	
3.77500033" "	0.896764994	
3.32500029" "	0.895362973	
2.02500033" "	0.891874015	
**7.17500019" "**	**0.876617014**	Acetaminophen derivates
3.17500019" "	0.872853994	
2.62500024" "	0.853223026	
2.42500019" "	0.848438978	
2.72500038" "	0.846161008	
0.97500026" "	0.842419982	
1.62500024" "	0.831054986	
7.92500019" "	0.800468981	
1.22500026" "	0.784087002	
9.12500000" "	0.744544029	
4.37500048" "	0.733959973	
3.42500019" "	0.729426026	
0.72500026" "	0.721149981	
4.27500010" "	0.710471988	
2.52500033" "	0.709295988	
2.92500019" "	0.700329006	
8.47500038" "	0.656902015	
4.47500038" "	0.65635401	
4.22500038" "	0.654479027	
4.12500048" "	0.650339007	
7.52500057" "	0.640393972	
1.27500021" "	0.632025003	
1.82500029" "	0.629858971	
1.37500024" "	0.628601015	
2.22500038" "	0.617352009	
8.02500057" "	0.610783994	
4.17500019" "	0.609121025	
1.12500024" "	0.608013988	
8.07500076" "	0.589842021	
6.97500038" "	0.588469028	
0.87500024" "	0.580259979	
1.07500029" "	0.548677981	
7.07500029" "	0.546272993	
1.67500031" "	0.544571996	

3.37500024" "	0.526149988	
1.72500026" "	0.475430995	
1.77500021" "	0.474720001	
2.82500029" "	0.471480012	
2.37500024" "	0.471421003	
2.97500038" "	0.464751989	
3.47500038" "	0.460727006	
3.82500029" "	0.457672	
1.02500021" "	0.45728901	
**7.67500019" "**	**0.438383013**	Hippuric acid
2.77500033" "	0.430925995	
0.82500023" "	0.416177005	
2.07500029" "	0.414418012	
8.57500076" "	0.399253994	
8.87500000" "	0.382472992	
8.17500019" "	0.374899	
7.77500057" "	0.352337003	
6.92500019" "	0.351927012	
8.62500000" "	0.344518989	
8.12500000" "	0.342314005	
1.97500026" "	0.336214989	
6.62500048" "	0.332626998	
0.62500024"	0.331806988	
8.27500057" "	0.329948008	
0.77500021" "	0.323285013	
6.77500057" "	0.313609987	
8.22500038" "	0.312730014	
0.67500025" "	0.289485991	
6.87500048" "	0.289072007	
8.37500000" "	0.268164992	
6.82500029" "	0.260825008	
7.72500038" "	0.258183002	
6.52500010" "	0.239063993	
6.57500029" "	0.223114997	
8.52500057" "	0.214689001	
7.87500048" "	0.210011005	
6.72500038" "	0.190148994	
8.42500019" "	0.147286996	
6.67500019" "	0.139761001	
7.97500038" "	0.120927997	
8.77500057" "	0.120274998	
8.32500076" "	0.114081003	
8.72500038" "	0.090801403	
4.52500010" "	0.080738999	
9.72500038" "	0.080002204	
9.27500057" "	0.065201998	

8.67500019" "	0.048496801	
9.47500038" "	0.047562402	
9.77500057" "	0.047542401	
9.02500057" "	0.045168102	
9.57500076" "	0.037988201	
9.32500076" "	0.037558001	
9.22500038" "	0.037190098	
9.67500019" "	0.034524601	
9.07500076" "	0.033525299	
9.52500057" "	0.033387899	
8.92500019" "	0.0324503	
9.17500019" "	0.0316412	
9.37500000" "	0.0311174	
9.87500000" "	0.030564001	
9.62500000" "	0.029415799	
9.92500019" "	0.0270292	
9.82500076" "	0.026491901	
9.42500019" "	0.025451699	
8.97500038" "	0.0253853	
9.97500038" "	0.016777501	

VIP for PLS-DA regarding total FIM

Primary ID (chemical shift)	VIP	Targeted metabolites
**2.17500019" "**	**3.507910013**	Paracetamol (Acetaminophen) + Acetaminophen glucuronide
3.62500024" "	2.895889997	
1.92500031" "	2.57359004	
7.82500029" "	2.220020056	
**7.12500048" "**	**2.147399902**	Acetaminophen derivates
7.32500029" "	2.137599945	
3.97500038" "	2.033760071	
3.27500033" "	2.015810013	
3.47500038" "	1.959319949	
3.02500033" "	1.807489991	
7.37500048" "	1.686609983	
7.57500029" "	1.655910015	
7.52500057" "	1.630929947	
1.42500031" "	1.552189946	
3.77500033" "	1.497400045	
3.57500029" "	1.406389952	
7.62500048" "	1.404539943	
0.87500024" "	1.396939993	

2.47500038" "	1.395370007	
3.92500019" "	1.387320042	
7.27500057" "	1.365689993	
3.42500019" "	1.359899998	
4.07500029" "	1.35698998	
3.72500038" "	1.332020044	
1.27500021" "	1.329550028	
2.57500029" "	1.326920033	
4.02500010" "	1.31408	
7.42500019" "	1.292050004	
4.42500019" "	1.28204	
2.22500038" "	1.272699952	
7.22500038" "	1.25030005	
3.87500024" "	1.228559971	
2.67500019" "	1.223109961	
3.12500024" "	1.22021997	
2.32500029" "	1.212139964	
7.47500038" "	1.193510056	
2.72500038" "	1.169039965	
4.17500019" "	1.159190059	
3.52500033" "	1.143460035	
3.67500019" "	1.107609987	
1.17500019" "	1.103770018	
3.37500024" "	1.091519952	
2.52500033" "	1.063269973	
4.27500010" "	1.048629999	
1.22500026" "	1.045660019	
3.22500038" "	1.038300037	
1.32500029" "	1.036730051	
1.87500024" "	1.034340024	
3.82500029" "	1.017740011	
2.27500033" "	1.009140015	
2.07500029" "	0.98634702	
8.82500076" "	0.984538972	
1.47500026" "	0.932119012	
1.72500026" "	0.922908008	
0.92500025" "	0.922538996	
6.92500019" "	0.911436021	
4.12500048" "	0.862652004	
1.67500031" "	0.857032001	
1.07500029" "	0.852024972	
1.62500024" "	0.850190997	
1.02500021" "	0.848508	
2.12500024" "	0.83664	
0.97500026" "	0.82872802	
**7.17500019" "**	**0.810796976**	Acetaminophen derivates

1.77500021" "	0.779514015	
1.57500029" "	0.757265985	
3.32500029" "	0.74387598	
8.07500076" "	0.737294972	
4.32500029" "	0.734041989	
2.97500038" "	0.718600988	
3.17500019" "	0.714731991	
7.72500038" "	0.712873995	
2.37500024" "	0.702413023	
9.12500000" "	0.698108971	
7.02500057" "	0.685755014	
1.97500026" "	0.685643971	
1.82500029" "	0.68244499	
4.37500048" "	0.68206799	
4.22500038" "	0.678451002	
2.92500019" "	0.675050974	
3.07500029" "	0.672038019	
7.92500019" "	0.671432972	
8.47500038" "	0.643477976	
2.02500033" "	0.633783996	
8.57500076" "	0.632566988	
6.87500048" "	0.630869985	
4.47500038" "	0.628924012	
1.37500024" "	0.625042975	
1.52500021" "	0.601720989	
7.67500019" "	0.594356	
2.82500029" "	0.574029982	
1.12500024" "	0.544685006	
2.42500019" "	0.533536971	
2.77500033" "	0.532441974	
2.62500024" "	0.512243986	
0.72500026" "	0.508642972	
8.62500000" "	0.423851013	
0.82500023" "	0.397287011	
8.32500076" "	0.365197003	
6.57500029" "	0.364463001	
8.22500038" "	0.356682986	
8.27500057" "	0.342691004	
0.77500021" "	0.334650993	
7.07500029" "	0.329358011	
2.87500024" "	0.325926006	
6.97500038" "	0.323323011	
8.87500000" "	0.313636005	
0.67500025" "	0.306818008	
0.62500024"	0.280856997	
6.82500029" "	0.243205994	

8.37500000" "	0.204136997	
6.77500057" "	0.192056	
7.87500048" "	0.189191997	
6.52500010" "	0.186387002	
7.97500038" "	0.183009997	
7.77500057" "	0.171222001	
6.67500019" "	0.163661003	
8.42500019" "	0.150803998	
8.52500057" "	0.141563997	
6.72500038" "	0.123822004	
6.62500048" "	0.102707997	
8.67500019" "	0.080725901	
8.97500038" "	0.0647965	
9.82500076" "	0.057742	
8.77500057" "	0.0577267	
9.22500038" "	0.056444898	
8.02500057" "	0.0549049	
9.07500076" "	0.0523431	
9.27500057" "	0.051940601	
9.92500019" "	0.050994098	
9.02500057" "	0.050936699	
8.17500019" "	0.049567401	
9.52500057" "	0.048691101	
9.97500038" "	0.048231501	
9.87500000" "	0.048103701	
9.77500057" "	0.045664798	
9.42500019" "	0.043876499	
9.62500000" "	0.0438172	
9.47500038" "	0.043655101	
8.92500019" "	0.043543	
9.32500076" "	0.043239001	
8.12500000" "	0.043184999	
9.17500019" "	0.041376401	
9.37500000" "	0.040614299	
9.57500076" "	0.040134799	
8.72500038" "	0.038870499	
9.67500019" "	0.038605802	
9.72500038" "	0.030107301	
4.52500010" "	0.0256663	

VIP for PLS-DA regarding motor component of FIM

Primary ID (chemical shift)	VIP	Targeted metabolites
**2.17500019" "**	**3.540280104**	Paracetamol (Acetaminophen) + Acetaminophen glucuronide
3.62500024" "	3.164220095	
**7.12500048" "**	**2.706029892**	Acetaminophen derivates
3.27500033" "	2.573440075	
4.07500029" "	2.467999935	
3.97500038" "	2.323559999	
3.02500033" "	2.307369947	
7.82500029" "	2.286619902	
7.32500029" "	2.238549948	
**1.92500031" "**	**1.947749972**	Acetate
**3.57500029" "**	**1.895810008**	Glycine
2.67500019" "	1.870460033	
7.57500029" "	1.669399977	
7.37500048" "	1.669059992	
7.52500057" "	1.64388001	
8.82500076" "	1.463950038	
0.87500024" "	1.456159949	
3.87500024" "	1.446959972	
7.62500048" "	1.441059947	
4.42500019" "	1.38530004	
3.32500029" "	1.357059956	
**7.17500019" "**	**1.347200036**	Acetaminophen derivates
2.12500024" "	1.308109999	
2.57500029" "	1.302189946	
3.77500033" "	1.301290035	
8.07500076" "	1.272570014	
4.02500010" "	1.263440013	
2.52500033" "	1.250480056	
3.22500038" "	1.201089978	
3.72500038" "	1.161170006	
1.27500021" "	1.142920017	
3.92500019" "	1.118489981	
2.87500024" "	1.070240021	
1.42500031" "	1.070160031	
3.52500033" "	1.055189967	
2.47500038" "	1.046519995	
3.82500029" "	1.023939967	
9.12500000" "	1.000180006	
7.02500057" "	0.992079973	
3.12500024" "	0.965856016	

6.97500038" "	0.924398005	
3.67500019" "	0.916547	
4.32500029" "	0.907444	
2.32500029" "	0.896317005	
7.42500019" "	0.84702599	
3.07500029" "	0.837459028	
1.32500029" "	0.831602991	
1.22500026" "	0.829024971	
3.17500019" "	0.827152014	
2.82500029" "	0.810347021	
4.27500010" "	0.795050979	
1.02500021" "	0.794301987	
1.07500029" "	0.786082983	
7.07500029" "	0.778559983	
3.37500024" "	0.768333018	
4.47500038" "	0.755715013	
1.17500019" "	0.749069989	
3.42500019" "	0.744826019	
0.92500025" "	0.737303019	
7.47500038" "	0.725162029	
7.97500038" "	0.722177982	
8.57500076" "	0.716573	
4.17500019" "	0.698027015	
1.47500026" "	0.697835028	
2.42500019" "	0.686851978	
0.72500026" "	0.680082023	
2.77500033" "	0.679543018	
**2.22500038" "**	**0.665754974**	Acetone
2.37500024" "	0.653419971	
4.12500048" "	0.641063988	
1.87500024" "	0.638963997	
8.47500038" "	0.622466981	
2.62500024" "	0.61709702	
1.82500029" "	0.612757027	
7.67500019" "	0.610632002	
3.47500038" "	0.605764985	
2.97500038" "	0.602792025	
2.02500033" "	0.602522016	
1.62500024" "	0.592383027	
7.72500038" "	0.58287698	
1.52500021" "	0.577071011	
6.82500029" "	0.553602993	
4.37500048" "	0.549178004	
1.37500024" "	0.540980995	
2.92500019" "	0.532335997	
4.22500038" "	0.511853993	

1.67500031" "	0.486193001	
7.22500038" "	0.484183013	
1.57500029" "	0.483810991	
0.97500026" "	0.483559996	
1.77500021" "	0.475443989	
6.87500048" "	0.450325012	
8.62500000" "	0.433279008	
2.07500029" "	0.431243986	
7.27500057" "	0.427237988	
8.87500000" "	0.422962993	
7.77500057" "	0.416563988	
2.27500033" "	0.412259996	
6.62500048" "	0.399426997	
1.72500026" "	0.397323012	
6.77500057" "	0.39261201	
1.12500024" "	0.382423997	
8.17500019" "	0.37281099	
**2.72500038" "**	**0.367215991**	Dimethylamine
7.87500048" "	0.338402987	
0.82500023" "	0.333602995	
8.37500000" "	0.326135993	
0.62500024"	0.319480002	
6.57500029" "	0.308353007	
6.52500010" "	0.306014001	
0.67500025" "	0.304387987	
8.52500057" "	0.303461999	
8.22500038" "	0.300437003	
6.92500019" "	0.298218995	
7.92500019" "	0.288340986	
1.97500026" "	0.281796992	
6.67500019" "	0.279828995	
0.77500021" "	0.262273997	
6.72500038" "	0.257734001	
8.32500076" "	0.257587999	
8.27500057" "	0.218898997	
8.02500057" "	0.214447007	
8.12500000" "	0.206196994	
8.42500019" "	0.165461004	
8.67500019" "	0.132401004	
9.02500057" "	0.115324996	
8.77500057" "	0.109944999	
9.72500038" "	0.108014002	
8.97500038" "	0.100500003	
9.27500057" "	0.096881799	
8.92500019" "	0.093246996	
9.67500019" "	0.092921101	

9.97500038" "	0.0894325	
8.72500038" "	0.088609204	
9.22500038" "	0.087710597	
9.82500076" "	0.085960403	
9.17500019" "	0.085089602	
9.87500000" "	0.085039802	
9.07500076" "	0.084417097	
9.42500019" "	0.081428401	
9.62500000" "	0.076260403	
9.37500000" "	0.075103603	
9.92500019" "	0.0707964	
9.52500057" "	0.069924198	
9.77500057" "	0.068051398	
9.32500076" "	0.067642398	
9.57500076" "	0.067051597	
9.47500038" "	0.0628362	
4.52500010" "	0.050185699	

VIP for PLS-DA regarding cognitive component of FIM

Primary ID (chemical shift)	VIP	Targeted metabolites
**2.17500019" "**	**3.580670118**	Paracetamol (Acetaminophen) + Acetaminophen glucuronide
3.27500033" "	3.120130062	
3.62500024" "	2.989099979	
**7.12500048" "**	**2.876940012**	Acetaminophen derivates
4.07500029" "	2.445760012	
7.32500029" "	2.128540039	
**7.17500019" "**	**2.019740105**	Acetaminophen derivates
3.02500033" "	2.00109005	
4.02500010" "	1.971529961	
3.87500024" "	1.906350017	
1.92500031" "	1.833989978	
2.12500024" "	1.819540024	
7.82500029" "	1.796790004	
3.97500038" "	1.785560012	
7.22500038" "	1.718140006	
3.77500033" "	1.674640059	
7.37500048" "	1.636000037	
4.42500019" "	1.60066998	
7.57500029" "	1.496700048	
3.92500019" "	1.38609004	

7.92500019" "	1.316509962	
3.17500019" "	1.300420046	
**2.22500038" "**	**1.22109997 3**	Acetone
3.72500038" "	1.21239996	
7.47500038" "	1.189399958	
**2.72500038" "**	**1.12205004 7**	Dimethylamine
7.27500057" "	1.077280045	
2.87500024" "	1.062770009	
7.62500048" "	1.054219961	
3.42500019" "	1.052209973	
3.47500038" "	1.045979977	
1.17500019" "	1.036749959	
1.42500031" "	1.016250014	
3.52500033" "	1.015699983	
3.37500024" "	0.979201019	
3.07500029" "	0.972541988	
3.82500029" "	0.956556976	
0.87500024" "	0.953836024	
1.87500024" "	0.94491601	
6.62500048" "	0.942669988	
8.82500076" "	0.917770982	
4.17500019" "	0.914976001	
7.42500019" "	0.910874009	
3.57500029" "	0.882044971	
2.57500029" "	0.869125009	
1.32500029" "	0.850059986	
2.27500033" "	0.849956989	
0.97500026" "	0.833805978	
2.67500019" "	0.829720974	
1.22500026" "	0.818176985	
9.12500000" "	0.804977	
3.67500019" "	0.803331017	
4.12500048" "	0.798340976	
2.62500024" "	0.798174977	
1.72500026" "	0.790269017	
1.67500031" "	0.776252985	
7.52500057" "	0.769765973	
2.52500033" "	0.756511986	
4.37500048" "	0.752882004	
3.12500024" "	0.742497027	
3.22500038" "	0.739880979	
6.67500019" "	0.734220982	
2.07500029" "	0.728034973	
1.77500021" "	0.721665025	
8.87500000" "	0.697538972	

1.62500024" "	0.694299996	
1.47500026" "	0.685970008	
2.32500029" "	0.676155984	
8.02500057" "	0.675288975	
1.02500021" "	0.674024999	
4.22500038" "	0.666764975	
1.57500029" "	0.663012981	
6.92500019" "	0.659008026	
1.12500024" "	0.658065021	
2.37500024" "	0.649703026	
4.47500038" "	0.646278977	
1.27500021" "	0.639084995	
2.92500019" "	0.639055014	
4.32500029" "	0.634131014	
2.47500038" "	0.632076979	
1.82500029" "	0.62866497	
2.97500038" "	0.619919002	
1.97500026" "	0.616255999	
2.82500029" "	0.599489987	
7.02500057" "	0.598078012	
2.77500033" "	0.574346006	
7.07500029" "	0.566619992	
0.92500025" "	0.564047992	
3.32500029" "	0.560163975	
1.07500029" "	0.559525013	
1.37500024" "	0.539552987	
7.67500019" "	0.536579013	
6.82500029" "	0.529246986	
4.27500010" "	0.516021013	
7.97500038" "	0.507849991	
1.52500021" "	0.501192987	
6.57500029" "	0.485552996	
2.42500019" "	0.482279003	
8.07500076" "	0.473531991	
2.02500033" "	0.468286991	
6.52500010" "	0.450183988	
0.77500021" "	0.448561013	
6.72500038" "	0.444444001	
0.72500026" "	0.439058006	
0.82500023" "	0.388603002	
7.77500057" "	0.385067999	
8.47500038" "	0.382245988	
8.57500076" "	0.380443007	
8.17500019" "	0.379078001	
7.72500038" "	0.372675002	
8.62500000" "	0.365193993	

0.67500025" "	0.363397002	
6.87500048" "	0.361054987	
6.77500057" "	0.343887001	
0.62500024"	0.322932988	
8.12500000" "	0.279980004	
8.52500057" "	0.246543005	
8.37500000" "	0.234124005	
6.97500038" "	0.198933005	
8.22500038" "	0.191062003	
7.87500048" "	0.179527	
8.32500076" "	0.168852001	
8.27500057" "	0.166382998	
8.67500019" "	0.118749	
9.17500019" "	0.101682	
9.97500038" "	0.084938802	
8.92500019" "	0.079984598	
9.37500000" "	0.075677097	
9.47500038" "	0.071273603	
9.62500000" "	0.070294097	
9.42500019" "	0.070078	
8.77500057" "	0.068929501	
9.87500000" "	0.068564601	
8.72500038" "	0.0683439	
9.57500076" "	0.068337001	
9.07500076" "	0.0666508	
9.77500057" "	0.064819098	
9.67500019" "	0.061509501	
9.92500019" "	0.0606381	
9.32500076" "	0.060236402	
9.02500057" "	0.056683999	
8.97500038" "	0.0558246	
9.82500076" "	0.055767801	
9.52500057" "	0.055245999	
9.27500057" "	0.0551618	
9.72500038" "	0.048636999	
9.22500038" "	0.0460383	
4.52500010" "	0.044241302	
8.42500019" "	0.042750999	

VIP for PLS-DA regarding SPPB at baseline

Primary ID (chemical shift)	VIP	Targeted metabolites

**2.17500019" "**	**3.512909889**	Paracetamol (Acetaminophen) + Acetaminophen glucuronide
3.62500024" "	2.815459967	
3.27500033" "	2.596070051	
4.07500029" "	2.400540113	
3.77500033" "	2.246860027	
7.12500048" "	2.225270033	
7.82500029" "	2.221560001	
7.32500029" "	2.199219942	
3.87500024" "	2.149600029	
3.97500038" "	2.070549965	
2.67500019" "	2.01748991	
4.02500010" "	2.000869989	
3.82500029" "	1.882419944	
7.37500048" "	1.854269981	
3.72500038" "	1.827110052	
7.57500029" "	1.667490005	
3.92500019" "	1.649359941	
3.52500033" "	1.584100008	
3.57500029" "	1.533220053	
3.22500038" "	1.506680012	
7.17500019" "	1.505849957	
7.62500048" "	1.421370029	
7.52500057" "	1.420379996	
3.02500033" "	1.413159966	
3.67500019" "	1.398100019	
**1.92500031" "**	**1.372230053**	Acetate
0.87500024" "	1.334879994	
8.82500076" "	1.33265996	
4.42500019" "	1.307279944	
7.02500057" "	1.277809978	
1.42500031" "	1.210500002	
1.17500019" "	1.181879997	
**1.37500024" "**	**1.167260051**	Lactate
1.22500026" "	1.148399949	
1.27500021" "	1.101240039	
7.27500057" "	1.069120049	
6.97500038" "	1.057010055	
1.07500029" "	1.017740011	
2.57500029" "	0.996105015	
1.52500021" "	0.989895999	
2.52500033" "	0.974425972	
9.12500000" "	0.892669022	
3.42500019" "	0.891768992	
2.87500024" "	0.871680021	

7.77500057" "	0.865637004	
3.07500029" "	0.858089983	
1.32500029" "	0.831487	
**2.22500038" "**	**0.815463006**	Acetone
2.47500038" "	0.764791012	
3.32500029" "	0.763934016	
4.32500029" "	0.755107999	
2.32500029" "	0.741243005	
2.92500019" "	0.739591002	
7.07500029" "	0.738407016	
3.37500024" "	0.738063991	
7.42500019" "	0.720871985	
3.17500019" "	0.712769985	
8.07500076" "	0.700515985	
4.47500038" "	0.690396011	
3.47500038" "	0.68800801	
7.47500038" "	0.682518005	
8.57500076" "	0.666337013	
4.27500010" "	0.657073021	
2.62500024" "	0.650548995	
6.87500048" "	0.64408499	
2.72500038" "	0.641593993	
0.72500026" "	0.631887019	
3.12500024" "	0.627487004	
4.17500019" "	0.626119971	
4.12500048" "	0.623179972	
**1.47500026" "**	**0.609432995**	Alanine
7.22500038" "	0.606330991	
2.02500033" "	0.601077974	
2.12500024" "	0.592585027	
2.42500019" "	0.581004977	
4.22500038" "	0.574449003	
4.37500048" "	0.562601984	
1.02500021" "	0.560297012	
2.82500029" "	0.559713006	
7.97500038" "	0.544098973	
8.22500038" "	0.542212009	
1.97500026" "	0.542168975	
8.02500057" "	0.533838987	
1.67500031" "	0.516384006	
1.82500029" "	0.499345988	
2.07500029" "	0.492547989	
7.67500019" "	0.479490995	
2.37500024" "	0.470099002	
0.97500026" "	0.468342006	

1.72500026" "	0.454252005	
1.87500024" "	0.429001004	
1.62500024" "	0.422419995	
2.77500033" "	0.410780996	
0.92500025" "	0.404621005	
1.57500029" "	0.403654993	
7.72500038" "	0.399958014	
8.62500000" "	0.391770989	
2.97500038" "	0.388238013	
1.12500024" "	0.362291008	
1.77500021" "	0.361378014	
7.92500019" "	0.342976004	
6.82500029" "	0.33495301	
8.87500000" "	0.306769013	
0.82500023" "	0.301243991	
6.92500019" "	0.288818002	
8.27500057" "	0.255508006	
0.77500021" "	0.242476001	
8.32500076" "	0.239109993	
0.67500025" "	0.232228994	
0.62500024"	0.229100004	
2.27500033" "	0.222666994	
6.62500048" "	0.215443999	
8.52500057" "	0.205983996	
6.77500057" "	0.181765005	
8.12500000" "	0.177134007	
8.47500038" "	0.173688993	
8.37500000" "	0.168236002	
7.87500048" "	0.166931003	
6.67500019" "	0.166602999	
9.72500038" "	0.139479995	
8.77500057" "	0.138460994	
6.72500038" "	0.136855006	
6.57500029" "	0.107058004	
9.82500076" "	0.101692997	
9.07500076" "	0.098023199	
8.17500019" "	0.0966281	
6.52500010" "	0.095339902	
9.77500057" "	0.091663398	
9.92500019" "	0.087485597	
9.22500038" "	0.0857803	
9.52500057" "	0.080743998	
9.87500000" "	0.076461099	
9.62500000" "	0.076252401	
8.72500038" "	0.074013397	
9.67500019" "	0.072376303	

9.32500076" "	0.072365902	
9.27500057" "	0.0679399	
9.57500076" "	0.065350302	
4.52500010" "	0.063555896	
8.67500019" "	0.0601914	
9.97500038" "	0.059533998	
9.37500000" "	0.059182301	
8.97500038" "	0.055453699	
8.92500019" "	0.055011801	
9.02500057" "	0.054043699	
9.42500019" "	0.052002799	
8.42500019" "	0.0471517	
9.47500038" "	0.046968799	
9.17500019" "	0.0388784	

VIP for PLS-DA regarding SPPB after 12 months

Primary ID (chemical shift)	VIP	Targeted metabolites
4.07500029" "	3.433180094	
**3.27500033" "**	**3.269949913**	TMAO
2.17500019" "	2.837589979	
7.82500029" "	2.326329947	
7.52500057" "	2.282910109	
7.17500019" "	2.20314002	
7.12500048" "	2.202950001	
2.67500019" "	2.023780107	
3.22500038" "	1.941140056	
8.82500076" "	1.932309985	
3.97500038" "	1.86135006	
4.42500019" "	1.837069988	
3.57500029" "	1.817579985	
4.02500010" "	1.726169944	
2.87500024" "	1.627320051	
3.12500024" "	1.618720055	
1.87500024" "	1.594799995	
**7.62500048" "**	**1.590489984**	Hippuric acid
3.72500038" "	1.562039971	
3.62500024" "	1.526039958	
2.52500033" "	1.450109959	
7.37500048" "	1.435490012	
7.32500029" "	1.426010013	
0.87500024" "	1.396839976	
9.12500000" "	1.360780001	

4.27500010" "	1.336619973	
2.57500029" "	1.331959963	
1.27500021" "	1.304810047	
1.42500031" "	1.197999954	
7.57500029" "	1.13301003	
2.32500029" "	1.125509977	
2.47500038" "	1.118489981	
3.07500029" "	1.111829996	
3.52500033" "	1.107740045	
3.67500019" "	1.063949943	
2.42500019" "	1.056030035	
4.47500038" "	1.047000051	
2.12500024" "	1.034809947	
2.02500033" "	1.019330025	
3.02500033" "	0.98951	
7.92500019" "	0.988188028	
3.17500019" "	0.972037971	
**1.92500031" "**	**0.952609003**	Acetate
8.57500076" "	0.928424001	
7.02500057" "	0.922134995	
3.37500024" "	0.917999029	
4.32500029" "	0.859907985	
**2.22500038" "**	**0.855675995**	Acetone
3.87500024" "	0.84246701	
3.42500019" "	0.837758005	
8.07500076" "	0.826223016	
7.07500029" "	0.808574975	
4.22500038" "	0.801088989	
4.17500019" "	0.793448985	
4.12500048" "	0.790503979	
3.77500033" "	0.757955015	
7.27500057" "	0.756859004	
4.37500048" "	0.742093027	
1.07500029" "	0.730614007	
1.37500024" "	0.722832024	
3.32500029" "	0.691488981	
6.97500038" "	0.690173984	
1.17500019" "	0.675261974	
1.52500021" "	0.674032986	
1.47500026" "	0.658580005	
2.97500038" "	0.651938975	
2.92500019" "	0.625930011	
2.27500033" "	0.623319983	
2.77500033" "	0.619596004	
1.22500026" "	0.616451025	
7.47500038" "	0.615541995	

3.82500029" "	0.613763988	
1.02500021" "	0.604955971	
**1.32500029" "**	**0.593318999**	Lactate
2.37500024" "	0.586853981	
8.17500019" "	0.586709976	
6.82500029" "	0.584164023	
**7.67500019" "**	**0.579671979**	
1.72500026" "	0.566438973	
1.62500024" "	0.554705977	
2.72500038" "	0.550706983	
3.92500019" "	0.536671996	
2.07500029" "	0.529794991	
1.82500029" "	0.528501987	
6.62500048" "	0.523881972	
7.42500019" "	0.515685976	
6.87500048" "	0.511116028	
0.92500025" "	0.504585981	
1.67500031" "	0.488467008	
7.77500057" "	0.482813001	
8.22500038" "	0.480168015	
7.87500048" "	0.473710001	
6.92500019" "	0.461053014	
1.77500021" "	0.457747012	
8.87500000" "	0.457242012	
8.47500038" "	0.451622993	
8.37500000" "	0.440632999	
1.57500029" "	0.417923987	
1.97500026" "	0.408549011	
2.82500029" "	0.408241004	
6.77500057" "	0.390648007	
3.47500038" "	0.383183002	
**0.97500026" "**	**0.382909**	Valine
8.62500000" "	0.373353004	
6.72500038" "	0.365529001	
7.22500038" "	0.364944994	
0.72500026" "	0.360915989	
8.27500057" "	0.356056988	
7.72500038" "	0.315138996	
6.67500019" "	0.313432008	
8.52500057" "	0.304044008	
6.57500029" "	0.295473993	
0.82500023" "	0.292849988	
7.97500038" "	0.291307002	
0.67500025" "	0.267298013	
0.62500024"	0.26086399	
2.62500024" "	0.254456013	

0.77500021" "	0.237210006	
6.52500010" "	0.231463999	
8.32500076" "	0.205871001	
8.12500000" "	0.205523998	
1.12500024" "	0.199608997	
8.42500019" "	0.175608993	
8.02500057" "	0.144948006	
8.67500019" "	0.111160003	
8.77500057" "	0.107490003	
9.72500038" "	0.102008	
9.02500057" "	0.090320602	
9.07500076" "	0.077213898	
9.82500076" "	0.076349102	
4.52500010" "	0.075899497	
9.77500057" "	0.070663698	
9.92500019" "	0.061778601	
9.27500057" "	0.061314501	
8.97500038" "	0.058582202	
8.72500038" "	0.0576168	
8.92500019" "	0.054319501	
9.52500057" "	0.050620198	
9.17500019" "	0.047325801	
9.97500038" "	0.046857402	
9.87500000" "	0.046033502	
9.22500038" "	0.045117699	
9.32500076" "	0.043583799	
9.67500019" "	0.043511599	
9.42500019" "	0.036835201	
9.47500038" "	0.036793798	
9.37500000" "	0.0359479	
9.57500076" "	0.033355899	
9.62500000" "	0.033151899	
